# An Improved Dung Beetle Optimizer for the Twin Stacker Cranes’ Scheduling Problem

**DOI:** 10.3390/biomimetics9110683

**Published:** 2024-11-07

**Authors:** Yidong Chen, Jinghua Li, Lei Zhou, Dening Song, Boxin Yang

**Affiliations:** 1 College of Shipbuilding Engineering, Harbin Engineering University, Harbin 150001, China; chenyidong@hrbeu.edu.cn; 2 College of Mechanical and Electrical Engineering, Harbin Engineering University, Harbin 150001, China; songdening@hrbeu.edu.cn (D.S.); yangboxin@hrbeu.edu.cn (B.Y.)

**Keywords:** shipbuilding material management, automated storage and retrieval system, improved dung beetle optimizer, twin stacker cranes’ scheduling, combinatorial optimization

## Abstract

In recent years, twin stacker crane units have been increasingly integrated into large automated storage and retrieval systems (AS/RSs) in shipyards to enhance operational efficiency. These common rail units often encounter conflicts, and the additional time costs incurred during collision avoidance significantly diminish AS/RS efficiency. Therefore, addressing the twin stacker cranes’ scheduling problem (TSSP) with a collision-free constraint is essential. This paper presents a novel approach to identifying and avoiding collisions by approximating the stacker crane’s trip trajectory as a triangular envelope. Utilizing the collision identification equation derived from this method, we express the collision-free constraint within the TSSP and formulate a mixed-integer programming model. Recognizing the multimodal characteristics of the TSSP objective function, we introduce the dung beetle optimizer (DBO), which excels in multimodal test functions, as the foundational framework for a heuristic optimizer aimed at large-scale TSSPs that are challenging for exact algorithms. To adapt the optimizer for bi-level programming problems like TSSPs, we propose a double-layer code mechanism and innovatively design a binary DBO for the binary layer. Additionally, we incorporate several components, including a hybrid initialization strategy, a Cauchy–Gaussian mixture distribution neighborhood search strategy, and a velocity revision strategy based on continuous space discretization, into the improved dung beetle optimizer (IDBO) to further enhance its performance. To validate the efficacy of the IDBO, we established a numerical experimental environment and generated a series of instances based on actual environmental parameters and operational conditions from an advanced AS/RS in southeastern China. Extensive comparative experiments on various scales and distributions demonstrate that the components of the IDBO significantly improve algorithm performance, yielding stable advantages over classical algorithms in solving TSSPs, with improvements exceeding 10%.

## 1. Introduction

The automated storage and retrieval system (AS/RS) is increasingly used in shipyard material management due to its advantages in space utilization and operational efficiency [1]. As productivity and market competitiveness in the shipbuilding industry improve, major shipyards are demanding higher storage and turnover capacities for their material preparation processes. Traditional AS/RS designs with single-stacker units are facing challenges as shelf heights and lengths continue to increase. For instance, the running time for a single stacker in the aisle is often excessive, leading to system downtime when spare stackers are not available for repairs. To address these issues, transitioning from single stacker units to common rail twin stacker units is essential for enhancing storage capacity in shipyards. However, twin stacker cranes operating on a common rail have a higher risk of collisions, which can result in additional costs for collision avoidance. These extra costs can undermine the operational efficiency that twin stackers are meant to provide. Therefore, addressing the twin stacker cranes’ scheduling problem (TSSP) is vital for the successful implementation and operation of twin stacker units in AS/RSs.

**Figure 1:**
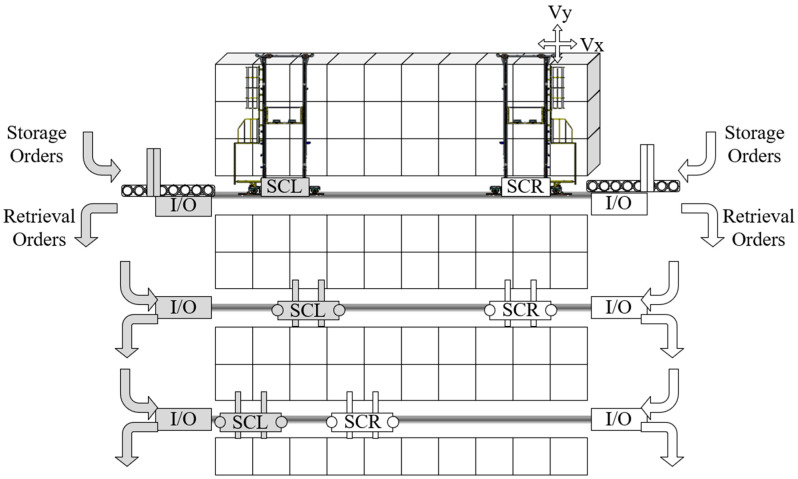
A typical TSSP in a shipyard.

**Figure 2:**
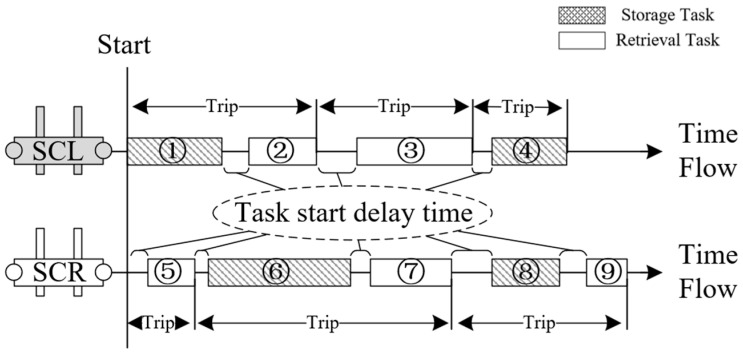
A scheduling scheme example for the TSSP.

The TSSP in a shipyard setting involves multiple twin stacker crane units completing various storage and retrieval tasks over a continuous work period, as illustrated in Figure 1. Upon arrival at the AS/RS, materials undergo a unified quality inspection before they can be stored. The storage locations for all materials are predetermined. The on-site production groups will make appointments to these materials in the form of order groups, known as “pallets”. Therefore, storage and retrieval tasks are static and free in shipyards, that is, the task objects are fixed, and they can be freely scheduled (including stacker crane allocation, task batching, task sorting, etc.). Figure 2 depicts a scheduling scheme for the TSSP, presented as a Gantt chart that illustrates the operations of the stacker crane on the left (SCL) and the stacker crane on the right (SCR).

**Figure 3:**
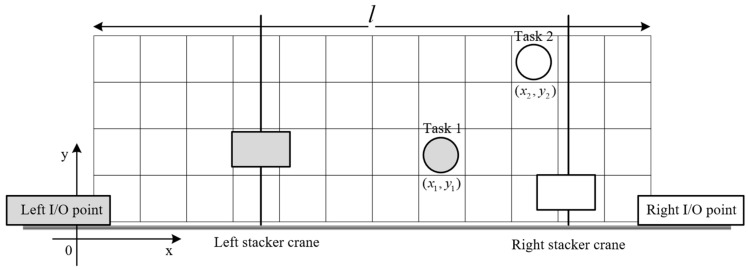
The sequential allocation of a two-task example.

**Figure 4:**
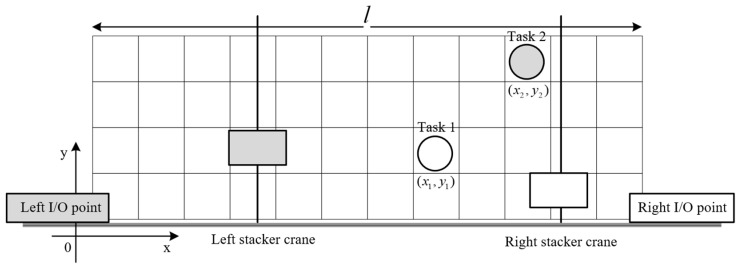
The reverse allocation of a two-task example.

In addition to AS/RS applications, common rail twin stacker crane units are frequently used in settings such as freight terminals and yards. In these environments, the logistics direction is typically fixed. For example, in a freight terminal, unloading orders must move from the seaside to the storage area, while boarding orders go from the storage area back to the seaside. Here, stacker crane movement is usually confined to one dimension. In contrast, the TSSP in shipyards requires allocation for the input and output (I/O) points of each order. Additionally, the vertical and horizontal movements of stacker cranes are critical factors that cannot be overlooked. This complexity results in larger solution spaces for shipyard TSSPs, making collision-free constraints more impactful on optimization objectives. To illustrate the characteristics of the TSSP in shipyards, consider two task points that overlap in time. Figure 3 and Figure 4 present two possible scheduling options for these tasks, in which the task point is assigned to the same-color stacker crane.

In Figure 3, there is no risk of conflict between the stacker cranes, which we refer to as sequential allocation. In contrast, Figure 4 presents a scenario with potential conflicts, termed reverse allocation.

To analyze the running times of the two scheduling options, we assume the total length of the shelf is l, the horizontal velocity of the stacker crane is $v_{x}$, the vertical velocity is $v_{y}$, and the coordinates of the task points are ${(x}_{1},y_{1})$ and ${(x}_{2},y_{2})$. The total running time t for the two stacker cranes can be calculated for each case as follows:

- For sequential allocation: $t_{1}=\max(\frac{x_{1}}{v_{x}},\frac{y_{1}}{v_{y}})+\max(\frac{{l-x}_{2}}{v_{x}},\frac{y_{2}}{v_{y}})$.
- For reverse allocation: $t_{2}=\max(\frac{{l-x}_{1}}{v_{x}},\frac{y_{1}}{v_{y}})+\max(\frac{x_{2}}{v_{x}},\frac{y_{2}}{v_{y}})$.

Assuming no additional criteria are introduced, a smaller t indicates a more efficient scheduling scheme. We can establish a sufficient condition for $t_{1}>t_{2}$ as $\left(x_{2}>x_{1}>\frac{l}{2})\&(\frac{x_{1}}{v_{x}}>\frac{y_{1}}{v_{y}})\&(\frac{x_{2}}{v_{x}}<\frac{y_{2}}{v_{y}}\right)$.

Under the above condition, reverse allocation is preferable. This suggests that overlapping task sequences between the two stacker cranes may lead to better scheduling optimization outcomes.

When the collision-free constraint is relaxed, the TSSP can be framed as a specialized multi-depot capacitated vehicle routing problem (MDCVRP). In this context, the left and right I/O points serve as distribution depots, each task point represents a customer, and the stacker crane functions as the vehicle. The load capacity of the stacker corresponds to the vehicle’s capacity, while distances between points are measured using Chebyshev distance. Since the TSSP can be reduced to the NP-hard MDCVRP, it is also NP-hard. Heuristic algorithms are known to be effective for approximating NP-hard problems. Thus, this study aims to develop a novel and effective scheduling optimizer that efficiently addresses collision-free constraints.

As shown in Figure 2, a TSSP’s task scheduling scheme involves several components, namely task allocation, task batching, task sorting, and the start delay time for each task. We refer to a scheduling scheme where all tasks have a start delay time of zero as the “sequence scheme”. Drawing inspiration from collision avoidance principles found in the related literature, it becomes clear that any collision-free scheduling scheme can be achieved by incorporating appropriate task start delays into the sequence scheme. By establishing a one-to-one mapping function between the sequence scheme and the collision-free scheduling scheme, the challenge of finding the optimal solution among collision-free schedules can be transformed into finding the optimal solution within the sequence schemes. This latter task is a common combinatorial optimization problem that is more straightforward to solve.

The main contributions of this paper can be summarized as follows:

1. This paper proposes a new idea to solve the TSSP, and according to this idea, a relaxation collision resolution approach by adding the trip start waiting time is proposed.
2. This paper proposes an improved dung beetle optimizer (IDBO) for large-scale TSSPs. We design a key component called a double-layer code mechanism, as well as other improvement strategies, to enhance the performance of the metaheuristic dung beetle optimizer (DBO).
3. The feasibility and applicability of the IDBO are verified through numerical experiments and enterprise case verification with the most powerful and classical metaheuristic algorithms.

The remainder of the paper is organized as follows: Section 2 gives a review of the related literature. Section 3 describes the problem and proposes a mixed-integer programming formulation based on a relaxed collision resolution method. Section 4 briefly introduces the basic DBO and gives the details of the IDBO. Computational results on numerous instances are reported in Section 5. Section 6 suggests approaches of research for enterprise decision-makers and management. Conclusions and future research directions are suggested in Section 7.

## 2. Related Work

The AS/RS not only has been widely used in practice but its corresponding planning issues also have been widely considered by the academic community. According to the surveys provided by Li, Y. [2], Azadeh, K. [3], and Boysen, N. [4], the TSSP belongs to the subproblem of the operation control and optimization problem in the AS/RS. Different from the single stacker crane scheduling optimization problem, the collision-free constraint brings stronger problem complexity and solving difficulty to the TSSP. Therefore, the handling of the collision-free constraint is the focus of TSSP research. Below, we will review the relevant works on crane scheduling considering the collision-free constraint to summarize existing methods and the gaps in them.

The scholars studying gantry crane scheduling problems first noticed the issue of the common rail equipment’s collisions. In the existing literature, the main methods to solve gantry cranes’ collision problems include approaches based on avoidance priority rules [5] (such as heuristic rules [6], small neighborhood 2-opt rules [7], deep learning rules based on digital twins [8], etc.) and approaches based on handshake regions [9,10] (such as fixed handshake regions [11], flexible handshake regions [12], handshake regions with capacity constraints [13], etc.). Due to the lack of global scheduling, there is a problem of poor optimization results of the approaches based on avoidance priority rules. The approaches of setting a handshake area can divide the activity area of two gantry cranes and provide a relay area for their task collaboration. Due to the fact that the gantry cranes’ scheduling optimization problems are typically simplified into one-dimensional scheduling problems, each berth has a certain depth and breadth, thereby ensuring the buffering capacity of the handshake area. However, the TSSP is a two-dimensional scheduling problem considering Chebyshev distances, where the shelves are usually single-depth, which is significantly different from the background of the gantry cranes’ scheduling optimization problem. Therefore, the approaches of setting a handshake area cannot be directly applied to the TSSP.

In addition to the quay crane scheduling problem, the multiple-reclaimers scheduling problem in the bulk terminal also has considered the conflict between two devices. For example, Angelelli [14] separates all stockpiles of each pad into two parts to avoid conflicts, while J. Xin [15] introduces a time–space network model to solve the conflict problem and proposed a two-level metaheuristic algorithm for solving it. Considering the complexity of reclaimer activities and their interactions, Burdett [16] carefully designed the prioritization method of reclaimers’ activities to enhance the quality of the solutions, and the conflict detection operator was designed during the iteration process of the algorithm to identify conflicting solutions and penalize their objective function values. However, there are essential differences between the reclaimer scheduling problem (RSP) and the TSSP. Firstly, the length of each pad of stockpiles is different in the RSP, while the storage location for each task in the TSSP is the same. Secondly, the movement of reclaimers is one-dimensional in the RSP, while that of stackers in the TSSP is two-dimensional. Thirdly, the reclaimers keep moving forward when reclaiming, while stacker cranes operate in situ. These makes the conclusions of the research on the RSP not applicable to the TSSP.

Even though the non-crossing constraint is widely investigated in the context of terminal scheduling problems, there are few approaches tackling this area in the context of AS/RS scheduling problems. As far as we know, Kung et al. [17] recognized the potential efficiency advantage of common rail stackers for the first time and pointed out that the collision problem was the key difficulty in common rail stackers’ application. To this end, they proposed an order cluster operation method to avoid collision by formulating resolution rules, thus transforming the common rail stackers’ scheduling problem into an order clusters’ clustering problem. However, this method is based on the premise that each cluster includes only one task from each stacker. In essence, it simplifies the collision problem through the rough discretization of operation time. Thus, the lower limit of the optimal makespan obtained by this method is too high. Inspired by the scheduling of the common rail gantry cranes at the wharf, some other scholars allocate tasks by setting a fixed [18] or dynamic [19,20] left–right interface on the shelf so as to ensure that the stackers are collision-free. However, this kind of order clustering method based on an interface is limited to the single optimization objective makespan, and it will be improper when including other optimization objectives.

The twin robot scheduling problem (TRSP) can be regarded as a special TSSP in which the stacker crane only moves horizontally. Erdogan et al. [21] first defined the TRSP and proved it to be NP-hard. For this problem, they constructed integer programming formulas based on time and priority indicators, respectively, to avoid the simultaneous execution of storage/retrieval requests with conflicting paths. A branch and bound algorithm and a heuristic algorithm were proposed for solving the TRSP. Boysen et al. [22] extended the TRSP by introducing the concept of a bottleneck robot and applied dynamic programming methods to determine a solution for the other robot with the collision-free constraint. Jaehn and Wiehl [23] further extended the TRSP by considering the types of storage/retrieval requests and makespan while designing precise and approximate algorithms to solve the problem. These works provide another approach to solve the TSSP, which is to first determine the task sequence of a high-priority stacker crane, and then combine it with the collision-free constraint to determine the task sequence of another stacker crane. However, this approach assumes that the task allocation of the stacker crane is completed in advance, which limits the flexibility of task scheduling in the twin stacker crane system.

In addition, some studies consider the variants of the stacker crane’s scheduling problem such as the dynamic arrival of orders [24,25] the identification method of stacker conflict when using deep reinforcement learning [26,27], the new mathematical model based on graph theory [28], and the use of digital twins to evaluate the energy consumption of operations [29].

To sum up, the existing approaches for the TSSP include the following four categories: 1. approaches based on avoidance priority rules; 2. approaches based on scheduling priority rules; 3. approaches based on time domain interface setting; 4. approaches based on space interface setting. However, the priority-based approaches reduce the system flexibility, and the interface-based approaches reduce the solution accuracy. Therefore, the TSSP is an under-researched area.

## 3. Problem Description and Modeling

In this section, we will describe the TSSP in detail and propose a mixed-integer programming model for the problem.

### 3.1. Problem Description

**Figure 5:**
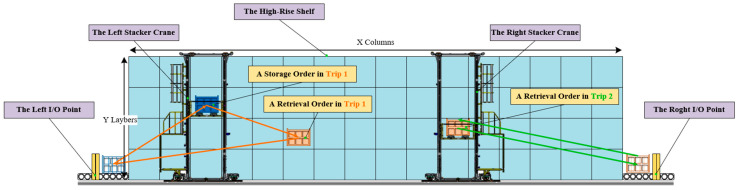
The twin common rail stacker system composition and an operational example.

As outfitting materials are the main storage objects in the shipyard’s AS/RS, we limited the research scenario of the TSSP to the outfitting warehouse in shipyards. As a production-oriented warehousing activity, shipyard AS/RS management has some differences from sales-oriented warehousing activities such as e-commerce AS/RSs. For example, its daily plan of storage and retrieval orders is relatively static, and each order corresponds to an established storage location. Additionally, after being retrieved, the outfitting orders need to be combined into the pallets required by the production department through a pallet picking activity. Due to the TSSP’s scheduling object being a static order pool, we can concentrate problem scenarios on a single shelf. Figure 5 describes the system composition of the TSSP and a simple operational example. The twin common rail stacker unit shown in the figure includes two single-load stackers sharing the same rail and a single-sided high-rise shelf with x columns and y layers. I/O points have been set up at both ends of the shelves for order handover. Considering capacity constraints, one stacker crane can complete a maximum of one storage order and one retrieval order during one trip in and out of the I/O point. Furthermore, when the task volume during the trip is two, the retrieval order must be subsequent to the storage order. For retrieval orders belonging to the same pallet but taken from different I/O points, it is necessary to additionally transport and gather them together. Therefore, the total cost of operation for a twin common rail stacker unit should not only consider the operation time span of stackers but also include the average preparation time of the corresponding pallets for each batch of retrieval orders and the additional time for gathering retrieval orders to the same end. In summary, the goal of the TSSP proposed in this paper is to obtain a twin stacker cranes’ scheduling scheme that minimizes the total cost of system parallel operation time, pallet preparation time, and pallet aggregation penalty time.

In order to facilitate problem analysis and model construction, we make the following common assumptions:

1. Only single-sided shelves are considered.
2. Storage orders can be received from any I/O point without considering the differences in I/O point allocation.
3. The horizontal and vertical movement speeds of the stacker crane are considered uniform without considering the acceleration and deceleration processes.
4. The stacker crane has the same operation time for loading and unloading each time, including interaction with the shelves and interaction with I/O points.
5. For the convenience of calculation, we normalize the horizontal and vertical distances in the model formulation section, that is, we use the horizontal/vertical movement distance of the stacker crane per unit time as the unit distance in the horizontal/vertical direction.

### 3.2. Collision Avoidance Approach

**Figure 6:**
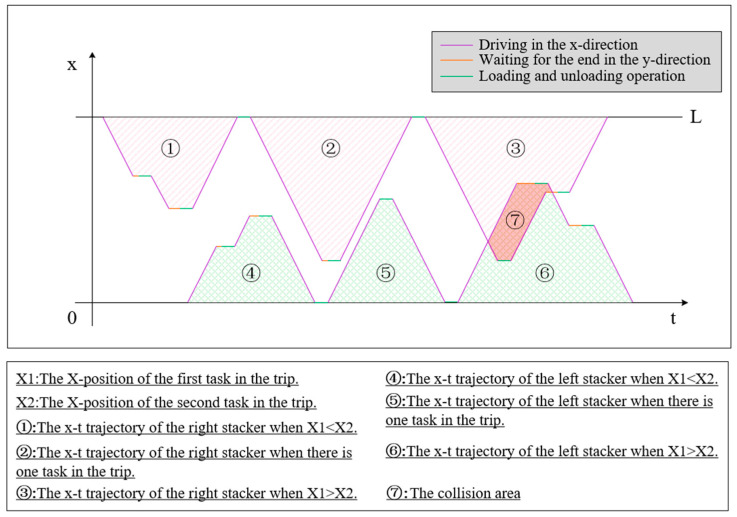
Possible x-t trajectories of stackers in one trip and an example of collision.

Since the collision of twin stackers is caused by the limited horizontal moving space, we show and discuss the collisions by using the x-t trajectory diagram, with the horizontal axis representing the time and the vertical axis representing the horizontal position of stackers. Figure 6 shows all the possible shapes of x-t trajectories corresponding to a single trip of the left stacker and the right stacker. An example of a collision is also presented in Figure 6. The intersection of trajectory lines means a collision will occur in this scheme. From the example, we can see that optimizing the scheduling scheme under the rigid collision-free constraint is extremely difficult and inefficient. It is difficult to express the collision-free constraint mathematically, and there is also the risk of missing the optimal solution. Therefore, this paper proposes a collision avoidance method by adding a delay time before each trip starts. This method “removes” the overlapping area by translating the trip trajectory block in the positive direction along the x-axis.

**Figure 7:**
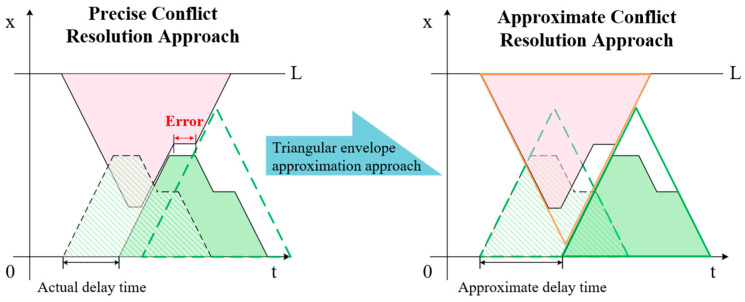
Replacing the multilateral trip trajectory block with a triangular envelope in the collision avoidance method.

Although any sequence scheme can be transformed into a collision-free scheduling scheme by adding the minimum trip start delay times, removing the overlaps between multilateral trip trajectory blocks remains a complex task. This task requires carefully listing all conflict situations and performing separate calculations for each situation, which is clearly unacceptable in the situation where the calculation cost needs to be accumulated many times per individual conversion. Therefore, when calculating the delay time in the collision avoidance method, we replace the multilateral trip trajectory block with a triangular envelope to simplify and unify the calculation formula, which can be named the triangular envelope approximation approach (TEAA), shown in Figure 7.

It cannot be denied that the TEAA requires some additional cost of time compared to the precise conflict resolution approach. Below, we will discuss the impact of this error. Even without discussing all conflict situations of trip trajectory blocks, it is easy to understand that there must be two adjacent tilted sides between the two blocks after using the precise approach to resolve the conflict, while after using the TEAA, the two adjacent tilted sides must be the outermost sides of the block. Therefore, the error generated by the TEAA is the horizontal distance from the adjacent sides to the outermost tilted side in the precise approach. In the example of Figure 7, this error is the length of the horizontal line segment labeled in the figure. Therefore, the upper limit of error generated by the TEAA for conflict resolution between each two trips is the sum of the time for a single loading/unloading operation of the stacker crane and the waiting time for the end of vertical moving. This means that the proportion of error is smaller when the task point is farther from the I/O point or the difference between the vertical distance between task points and the Chebyshev distance is smaller. Therefore, the error generated by the TEAA is acceptable in the context of long rails and the standard design and operation parameters of the AS/RS.

Assuming that the total length of the shelf is l, the stacker’s horizontal travel speed is $v_{x}$, the farthest distance between the left stacker and the left I/O point during the trip is $x_{1}$, the farthest distance between the right stacker and the right I/O point during the trip is $x_{2}$, and the two pending trips’ start and end times are $T_{1}^{s}$, $T_{1}^{e}$, $T_{2}^{s}$, and $T_{2}^{e}$, in that way, we can present the calculation process of the trips’ start delay times (the left stacker’s trip start delay time $\Delta t_{1}$ and the right stacker’s trip start delay time $\Delta t_{2}$) as Equations (1) and (2).

(1) $$\Delta t_{1}=\left\{\begin{array}{l} \max(0,\min(T_{2}^{e}-T_{1}^{s},T_{1}^{e}-T_{2}^{s})-l/v_{x}),T_{2}^{s}+T_{2}^{e}<T_{1}^{s}+T_{1}^{e}\wedge x_{1}+x_{2}>l\\ 0,T_{2}^{s}+T_{2}^{e}\geq T_{1}^{s}+T_{1}^{e}\\ 0,x_{1}+x_{2}\leq l \end{array}\right.$$

(2) $$\Delta t_{2}=\left\{\begin{array}{l} \max(0,\min(T_{2}^{e}-T_{1}^{s},T_{1}^{e}-T_{2}^{s})-l/v_{x}),T_{2}^{s}+T_{2}^{e}>T_{1}^{s}+T_{1}^{e}\wedge x_{1}+x_{2}>l\\ 0,T_{2}^{s}+T_{2}^{e}<T_{1}^{s}+T_{1}^{e}\\ 0,x_{1}+x_{2}\leq l \end{array}\right.$$

Obviously, Equations (1) and (2) can be easily transcoded into computational programs to serve any algorithm used for solving the TSSP.

The main idea of the collision avoidance approach described above is to simplify conflict resolution calculations by relaxing the spatiotemporal occupancy of each trip. Therefore, this approach can be more generally referred to as the collision avoidance approach based on relaxed trip trajectories.

For practical application needs, the TEAA was used to convert the sequence scheme into a collision-free scheduling scheme in the previous text. But it can also be used to modify any scheduling scheme to be collision-free. In this case, if each trip start delay time equals zero, it can be determined that this scheduling scheme satisfies the collision-free constraint.

Therefore, Equations (1) and (2) also contribute to formulating the mathematical model of the TSSP.

### 3.3. Mathematical Formulation

#### 3.3.1. Basic Notations

Besides the problem description, we formulate a mixed-integer programming model for the TSSP on the basis of referencing the mathematical model formulated in the vehicle routing problem with time windows. The TSSP’s mathematical model is defined on a directed graph G=(V,A), where V is the point set and A is the arc set. First, we specify the variables and parameters of the model in Table 1.

#### 3.3.2. Objective Function

The optimization objective of the TSSP is to minimize the total cost of stackers’ operation time, pallets’ preparation time, and sub-pallets’ aggregation penalty time. The objective function is modeled as Equations (3)–(6).

(3) $$\min z=f_{1}+\alpha f_{2}+\beta f_{3}$$

(4) $$f_{1}=\max(\sum_{k\in K}\sum_{j\in V}s_{ik}x_{ijk})$$

(5) $$f_{2}=\sum_{P_{n}\in J_{2}}(\underset{i\in P_{n}}{\max}(\sum_{k\in K}\sum_{j\in V}s_{ik}x_{ijk})-\underset{i\in P_{n}}{\min}(\sum_{k\in K}\sum_{j\in V}s_{ik}x_{ijk}))/N$$

(6) $$f_{3}=L\sum_{P_{n}\in J_{2}}\left|\sum_{i\in P_{n}}\sum_{k\in K^{L}}\sum_{j\in V}x_{ijk}-\sum_{i\in P_{n}}\sum_{k\in K^{R}}\sum_{j\in V}x_{ijk}\right|$$

$f_{1}$ represents the parallel operating span of the twin stackers. $f_{2}$ represents the average open time of the pallets, with the open time starting with the completion of the first order in the pallet and ending with the completion of the last order. $f_{3}$ means the extra transport time paid to gather the orders into the pallet together. α and β are weight factors, whose specific values are related to the actual application environment and the decision-making tendency. In this paper, they are set as α=1,β=0.2 according to the experience of shipyard managers.

#### 3.3.3. Constraints

The objective function z is subject to the following hard constraints:

- Constraint 1: Each task is executed only once, which can be guaranteed by Equations (7) and (8).

(7) $$\sum_{j\in V}\sum_{k\in K}x_{ijk}=1,\forall i\in J$$

(8) $$\sum_{i\in V}\sum_{k\in K}x_{ijk}=1,\forall j\in J$$

Equation (7) represents that the out-degree of any task point i in the directed graph G equals one, and Formula (8) represents that the in-degree of any task point j equals one.

- Constraint 2: The stacker must leave after completing a task point, which can be guaranteed by Equation (9).

(9) $$\sum_{i\in V}x_{ihk}=\sum_{j\in V}x_{hjk},\forall h\in J,k\in K$$

According to constraint 1, the values of $\sum_{i\in V}x_{ihk}$ and $\sum_{j\in V}x_{hjk}$ are both within the range of {0,1}; $\sum_{i\in V}x_{ihk}=1$ indicates that task point h and its adjacent predecessor point are in trip k. If $\sum_{i\in V}x_{ihk}=0$, task point h and its adjacent predecessor point are not entirely in trip k. $\sum_{j\in V}x_{hjk}=1$ indicates that task point h and its adjacent sequential point are in trip k. If $\sum_{j\in V}x_{hjk}=0$, task point h and its adjacent sequential point are not entirely in trip k. Therefore, Equation (9) ensures that every task point in any trip has an adjacent predecessor point and an adjacent sequential point, that is, there are no breakpoints in all trips.

- Constraint 3: Each trip starts from the I/O point, which can be guaranteed by Equation (10).

(10) $$\sum_{j\in V}x_{D_{k},j,k}=1,\forall k\in K$$

Equation (10) represents that in any trip, there is only one I/O point’s out-degree equaling one. Due to constraint 1 restricting the in-degree of all task points not equal to 0 and constraint 2 restricting all task points and their adjacent predecessor points to be in the same trips, all task points are not allowed to be the starting points of trips. Thus, Equation (9) can limit all trips to start from a unique I/O point.

- Constraint 4: Each trip ends at the I/O point, which can be guaranteed by Equation (11).

(11) $$\sum_{i\in V}x_{i,D_{k}^{\prime },k}=1,\forall k\in K$$

The explanation of Equation (11) is similar to that of Equation (10).

- Constraint 5: The time to leave a task point cannot be earlier than the completion time of this task point, and there should not be any sub-loops in the task sequence, which can be guaranteed by Equation (12).

(12) $$s_{ik}+t_{ij}+t_{ij}^{o}-s_{jk}\leq (1-x_{ijk})M,\forall i,j\in V,k\in K$$

When task point i is the adjacent predecessor point of task point j in trip k, $x_{ijk}=1$, and Equation (12) can be expressed as $s_{ik}+t_{ij}+t_{ij}^{o}\leq s_{jk}$, where $s_{ik}+t_{ij}+t_{ij}^{o}$ represents the completion time of task point j, constraint 5 is satisfied at this point.

When task point i is not the adjacent predecessor point of task point j in trip k, we only need to constrain the difference between task points’ completion time not to exceed the upper limit of the TSSP makespan (i.e., the symbol M).

M is a sufficiently large positive number. Here, we take the operation time of a single stacker to execute orders one by one as the value of M, which can be calculated as Equation (13).

(13) $$M=\max(\sum_{j\in J}(2t_{D_{1},j}+t_{j}^{o}),\sum_{j\in J}\left(2t_{D_{2},j}+t_{j}^{o}\right))$$

- Constraint 6: The start time of each trip cannot be earlier than the end time of the same stacker’s previous trip, which can be guaranteed by Equation (14).

(14) $$s_{ik}+t_{i,D_{k}^{\prime }}+t_{i,D_{k}^{\prime }}^{o}-s_{jh}\leq (2-\sum_{m\in V}x_{imk}-\sum_{n\in V}x_{jnh}+|h-k-1|)M,\forall i,j\in V,\forall h,k\in K^{L}or\;\forall h,k\in K^{R}$$

Equation (14) extends the expression of constraint 5 from the relationship between task points to the relationship between trips.

- Constraint 7: The storage and retrieval tasks within a single trip need to meet the stacker’s capacity constraint, which can be guaranteed by Equation (15).

(15) $$\sum_{i\in J_{1}}\sum_{j\in J_{1}}x_{ijk}+\sum_{i\in J_{2}}\sum_{j\in J_{2}}x_{ijk}+\sum_{i\in J_{2}}\sum_{j\in J_{1}}x_{ijk}=0,\forall k\in K$$

For a single-load stacker crane, the capacity constraint is equivalent to not allowing the following combinations of task points in all trips: (storage, storage), (retrieval, retrieval), and (retrieval, storage). Excluding these three combinations of task points corresponds to the following constraints: $\sum_{i\in J_{1}}\sum_{j\in J_{1}}x_{ijk}=0,\sum_{i\in J_{2}}\sum_{j\in J_{2}}x_{ijk}=0$ and $\sum_{i\in J_{2}}\sum_{j\in J_{1}}x_{ijk}=0$. Therefore, Equation (12) can ensure constraint 7.

- Constraint 8: Two stackers shall not collide during operation. According to the collision avoidance method proposed above, we can express the constraint in Equation (16).

(16) $$\min(\sum_{m\in V}s_{nh}x_{mnh}-\sum_{j\in V}s_{ik}x_{ijk},\sum_{i\in V}s_{jk}x_{ijk}-\sum_{n\in V}s_{mh}x_{mnh})<L^{t},\forall k\in K^{L},h\in K^{R}$$

In equation (16), $\sum_{m\in V}s_{nh}x_{mnh}$ represents the end time of the right stacker crane’s trip h, $\sum_{j\in V}s_{ik}x_{ijk}$ represents the start time of the left stacker crane’s trip k, $\sum_{i\in V}s_{jk}x_{ijk}$ represents the end time of the left stacker crane’s trip k, and $\sum_{n\in V}s_{mh}x_{mnh}$ represents the start time of the right stacker crane’s trip h.

- Constraint 9: take the start time of this order pool as the time origin, which can be guaranteed by Equation (17).

(17) $$s_{ik}\geq 0,\forall i\in V,k\in K$$

## 4. Improved Dung Beetle Optimizer Design for TSSP

The mathematical model of the TSSP formulated above is difficult to solve by CPLEX in a reasonable time under the condition of a large scale, so it is necessary to find a more rapid and practical algorithm for the TSSP. The DBO is a new optimization algorithm based on swarm intelligence proposed in 2022 [30]. It has the advantages of high accuracy, fast convergence, and good stability. The DBO has been widely used in path optimization [31,32,33,34], engineering design [35,36], data prediction [37,38,39], deep learning parameter optimization [40,41], and other issues.

The TSSP is a typical combinatorial optimization problem. Each scheduling scheme of the TSSP includes two parts, the stacker crane’s task allocation and task batching and sorting, among which the task allocation scheme has the main impact on the objective function value. Meanwhile, each task allocation scheme corresponds to multiple task batching and sorting. Thus, the TSSP has a multimodal characteristic in a certain sense. The test results of the basic DBO on CEC-BC-2017 functions demonstrate that it is superior to most other metaheuristic algorithms in solving multimodal, hybrid, and composition functions. In addition, its algorithm framework is relatively simple and easy to improve and expand. Specifically, the DBO contains non-interfering subpopulations with different iterative formulas, which can be made applicable to optimization problems with different characteristics by rewriting the position update formulas of these subpopulations or by adjusting the proportion of subpopulations. Therefore, it is a desirable method to apply the basic DBO to solve TSSPs after appropriate discretization and operator improvement.

### 4.1. Structure of Basic Dung Beetle Optimizer

Inspired by the various ecological activities of the dung beetle population in nature, the DBO divides the individual population into four subpopulations, namely ball-rolling dung beetles, breeding dung beetles, small dung beetles, and stealing dung beetles. Each subpopulation has its own independent location update rules.

(1) Ball-rolling dung beetles

Equation (18) imitates the ball-rolling process of the dung beetles guided by the sun when they do not encounter an obstacle. Equation (19) imitates the process of the dung beetle randomly selecting a new rolling direction through dance when encountering an obstacle.

(18) $$X_{i}(t+1)=X_{i}(t)+\alpha kX_{i}(t-1)+b\Delta x$$

(19) $$X_{i}(t+1)=X_{i}(t)+\tan\theta |X_{i}(t)-X_{i}(t-1)|$$

In Equation (18), $X_{i}(t)$ represents the position of individual i in generation t. α is the deflection coefficient affected by probability. When α=1, there is no deflection. When α=−1, it means deflection occurs in complex situations. $\Delta x=|x_{i}(t)-X^{w}|$ indicates the direction directed by the light source, where $X^{w}$ indicates the position of the global worst solution. k∈(0,0.2], b∈(0,1) are constant parameters, and the values are recommended as k=0.1,b=0.3 from the basic DBO. In Equation (19), θ∈(0,π) is the deflection angle randomly determined by the dung beetle through dance. When $\theta =\frac{\pi }{2}$, the position will not be updated in this generation.

(2) Breeding dung beetles

Equation (20) imitates the process of breeding dung beetles laying eggs randomly in the safe zone. The boundary of the safe zone is determined by Equation (21).

(20) $$B_{i}(t+1)=X^{*}+b_{1}(B_{i}(t)-Lb^{*})+b_{2}(B_{i}(t)-Ub^{*})$$

(21) $$\left\{\begin{array}{l} Lb^{*}=\max(X^{*}(1-R),Lb)\\ Ub^{*}=\min(X^{*}(1+R),Ub) \end{array}\right.$$

$Lb^{*}$ and $Ub^{*}$ represent the lower and upper bounds of the safe zone, Lb and Ub represent the lower and upper bounds of the variable definition field, and $X^{*}$ represents the local optimal solution. $R=1-t/T_{max}$ and $T_{max}$ represents the maximum number of iterations. $b_{1}$ and $b_{2}$ are independent random 1×D vectors, where D is the dimension of the problem.

(3) Small dung beetles

After birth, small dung beetles will forage in the best foraging area based on historical information. Equation (22) gives the boundary of the optimal foraging region. $Lb^{b}$ and $Ub^{b}$ represent the lower and upper bounds of the foraging region, and $X^{b}$ represents the global optimal solution.

(22) $$\left\{\begin{array}{l} Lb^{b}=\max(X^{b}(1-R),Lb)\\ Ub^{b}=\min(X^{b}(1+R),Ub) \end{array}\right.$$

In the foraging region, the individual dung beetle updates its position according to Equation (23) for foraging.

(23) $$X_{i}(t+1)=X_{i}(t)+C_{1}(X_{i}(t)-Lb^{b})+C_{2}(X_{i}(t)-Ub^{b})$$

$C_{1}$ is a 1×D random vector with normal distribution in each dimension and $C_{2}$ is a 1×D random vector with value range of (0,1) for each dimension.

(4) Stealing dung beetles

When dung beetles are short of food, they will steal dung balls from other dung beetles. Usually, the thieves will wander around the best foraging sites in search of theft opportunities. Inspired by this behavior, the basic DBO gave Equation (24) for updating the stealing dung beetles’ position.

(24) $$X_{i}(t+1)=X^{b}+sg(|X_{i}(t)-X^{*}|+|X_{i}(t)-X^{b}|)$$

s is a constant parameter and g is a 1×D random vector with normal distribution in each dimension.

### 4.2. Improved Dung Beetle Optimizer Design

The basic DBO was designed for continuous optimization problems. According to the no free lunch theorem [42], its performance advantage cannot be guaranteed in the combinatorial optimization problem. In addition, although the TSSP is a complex problem without an exact function shape, it must have typical multimodal characteristics as a special batch scheduling problem. Therefore, based on the characteristics of the TSSP, the improvement objectives of the basic DBO should include 1. having the adaptability of discrete optimization; 2. maintaining and improving the global search ability; and 3. improving the ability to jump out of the premature trap.

In the rest of this section, we give the design details of the IDBO for the TSSP. Section 4.2.1 introduces the double-layer code mechanism. We specifically designed a double-layer encoding and decoding mechanism that includes a continuous variable layer and a binary variable layer and innovatively provided a series of position update equations for a binary DBO based on the DBO framework. Section 4.2.2 introduces a hybrid initialization strategy to improve the quality of the initial population. In Section 4.2.3, we described a neighborhood search operator based on Cauchy–Gaussian mixture distribution. In Section 4.2.4, we proposed a velocity revising strategy during algorithm iterations to reduce the impact of optimization distortion when using metaheuristic algorithms to directly solve combinatorial optimization problems. Section 4.2.5 made some other improvements to some designs in the basic DBO that were not applicable to the TSSP.

#### 4.2.1. Double-Layer Code Mechanism

The TSSP involves the sorting and batching of orders. For such problems, continuous integers are usually used to represent the task sequence, and a special integer, such as “0”, is used to represent the separation point [43]. Although such explicit coding results are easy to understand, the unbalanced and independent information expression ability of each gene usually affects the application effect of metaheuristic algorithms in the TSSP and other sequence scheduling problems. In order to avoid this root problem that may damage the performance of the algorithm, this paper divides each solution’s code string into two layers, the continuous variable code string and the binary code string. Encoding, decoding, and updating methods based on this coded string structure are collectively referred to as the double-layer code mechanism.

#### Encoding Mechanism

**Figure 8:**
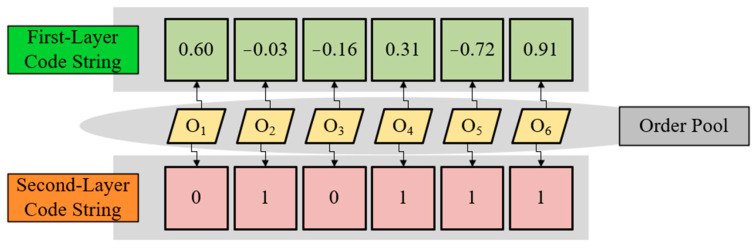
A double-layer encoding example of six orders.

Figure 8 shows a double-layer encoding example for a TSSP of six orders, in which each code locus corresponds to an order. Each code value in the first-layer code string represents the corresponding order’s distance to the origin in the time flow, while the second-layer code string reflects the stacker allocation of orders. Depending on the type of the code’s variable, we refer to the first layer as the continuous layer and the second layer as the binary layer. When an order is assigned to the left stacker, the corresponding second-layer code value equals zero; when assigned to the right stacker, the code value equals one. The example in Figure 8 corresponds to the task sequences SL={3,1},SR={5,2,4,6}, where SL represents the left stacker’s task sequence and SR represents the right stacker’s task sequence.

##### Decoding Mechanism

The process of calculating the fitness value from the double-layer code strings includes the following steps:

- Step 1: Transcode to stackers’ task sequences. By reading the information in the double-layer code string and tracing each task point on the time axis, the stacker task sequence can be easily obtained in chronological order.
- Step 2: Divide the trips. According to the task category of each order, find all the “storage-retrieval” combinations in the task sequences and divide them into the same trips, and then treat each remaining task as a separate trip. It is obvious that the optimal trip partition result for an individual is unique.
- Step 3: Calculate the trips’ start and end times. Starting from the first trip, the start and end times of each trip without considering collisions can be obtained by accumulating the Chebyshev travel times between task points in the trip and the operation time of each task point.
- Step 4: Revise the trips’ start and end times. Add the start delay time for each trip according to Equations (1) and (2).
- Step 5: Calculate the fitness value. Inspect the two stackers’ revised trip start and end schedules and take the maximum completion time of the stackers as F1. Then, check the ready time of retrieval orders in each pallet (take the time when the order is sent to the I/O point as the order ready time) and calculate the open time of the pallet with the earliest and latest order ready time. F2, the second part of the fitness value, can be obtained after taking the mean value of pallets’ open times. Finally, calculate the left and right I/O point distribution of orders in each pallet and calculate the sub-pallets’ aggregation penalty time F3.

**Figure 9:**
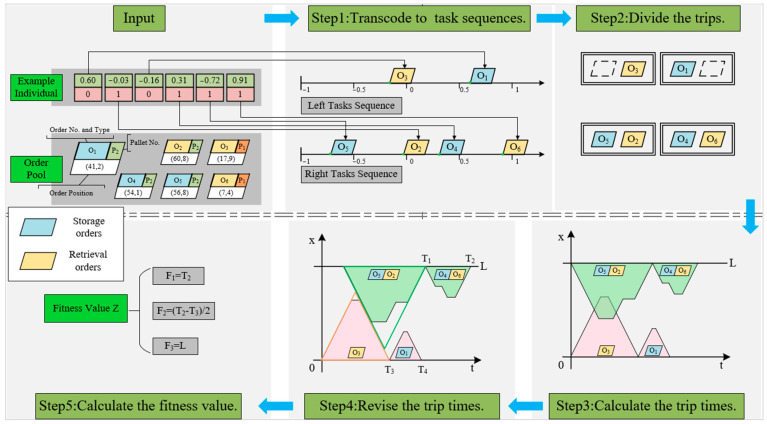
A double-layer decoding example of six orders.

Figure 9 illustrates the decoding process for an individual containing six orders.

It can be clearly seen from step 2 in Figure 9 that the double-layer code mechanism can effectively ensure the independence and unity of the code information’s expression during the algorithm iteration. For each task corresponding to each order, its position in the task sequences can be determined by its first-layer and second-layer code values, which is an aspect of independence. The other aspect of independence is that when we change any code’s value, it will not affect the expression of other codes. For example, when we change the first-layer code’s value, it will only cause the corresponding task point to move left or right in the task sequence, while changing the second-layer code’s value will only cause the task point to jump between two task sequences. The disturbance will not affect the position of other task points in the task sequence. In addition, due to the consistent variable range and independence of the codes in the same layer, it is easy to apply and adjust the individual update equations in various algorithms. This reflects the advantage of the unified code information of the double-layer code mechanism.

##### Code String Update Mechanism

In the double-layer structure, the continuous layer is still applicable to the original subpopulation position update equations, while the binary layer requires appropriate modifications to these equations.

Hamming weights and Hamming distances are common concepts used to measure the information difference between strings in informatics [44]. We introduce these two concepts into the second-layer code string update process, taking the Hamming weight $H(X_{i}(t),0)$ of the coding string as the individual’s position and the Hamming distance $H(X_{i}(t),X_{j}(t))$ between coding strings as the distance between individuals. Therefore, we give and explain the position update equations of subpopulations’ second-layer code strings as follows:

(1) Ball-rolling dung beetles

The position update mechanism of the ball-rolling individuals’ binary code strings is shown in Equation (25).

(25) $$H(X_{i}(t+1),X_{i}(t))=[kH(X_{i}(t-1),0)]\vee [bH(X_{i}(t),X^{w})]$$

$X_{i}(t)$ represents the current individual, $X_{i}(t-1)$ represents the previous generation individual, $X_{i}(t+1)$ represents the updated individual, $X^{w}$ represents the global worst individual, and round represents the rounding function.

The position update of the binary code string described by Equation (25) includes two steps. 1. Find all the different codes in $X_{i}(t)$ whose values are different from the corresponding loci in $X_{i}(t-1)$, then randomly select $\left[kH\left(X_{i}\left(t-1\right),0\right)\right]$ loci from them to flip. If $\left[kH\left(X_{i}\left(t-1\right),0\right)\right]$ exceeds the number of different codes, flip all the different codes. 2. Find all the codes in $X_{i}(t)$ whose values are the same as the corresponding loci in $X^{w}$, then randomly select $\left[bH\left(X_{i}\left(t\right),X^{w}\right)\right]$ loci from them to flip. If $\left[bH\left(X_{i}\left(t\right),X^{w}\right)\right]$ exceeds the number of same codes, flip all the same codes.

It should be noted that the code flipping in the two steps follows the logic OR gate, that is, as long as the flip needs to occur in either step, the final flip is carried out. In addition, as there is a certain randomness in the selection of code loci to flip, it is unnecessary to set a small probability event to update the position in the binary layer of the ball-rolling individual.

(2) Breeding dung beetles

The position update mechanism of the breeding individuals’ binary code strings is shown in Equations (26) and (27).

(26) $$H(X_{i}(t+1),0)=H(X^{*},0)-\mathrm{randi}(Ub^{*}-H(X_{i}(t),0))+\mathrm{randi}(H(X_{i}(t),0)-Lb^{*})$$

(27) $$\left\{\begin{array}{l} Lb^{*}=\max(H(X^{*},0)-R\frac{Dim}{2},0)\\ Ub^{*}=\min(H(X^{*},0)+R\frac{Dim}{2},Dim) \end{array}\right.$$

$X_{i}(t)$ represents the current individual, $X_{i}(t+1)$ represents the updated individual, $X^{*}$ represents the best individual in the current generation, $R=1-t/T_{max}$ represents the regional attenuation coefficient, Dim represents the problem dimension, randi⁡(x) represents the random rounding function within the range of [0,x], and −randi⁡(|x|) is taken if x<0.

The position update of the binary code string described by Equation (26) includes two steps. 1. Randomly select $\mathrm{randi}(Ub^{*}-H(X_{i}(t),0))$ code loci from all the loci with the value of “1” and $\mathrm{randi}(H(X_{i}(t),0)-Lb^{*})$ code loci from all the loci with the value of “0” in the local optimal individual to flip. If the number to flip exceeds the limit during each of the two flipping operations, flip all codes. 2. Check whether the Hamming distance of $X_{i}(t+1)$ after these two flipping operations exceeds the range specified by Equation (27) and restore the corresponding number of flips to ensure that it does not exceed the boundary. After determining the type and quantity, the restorage operation is carried out by random selection.

(3) Small dung beetles

The position update mechanism of the small dung beetles’ binary code strings is shown in Equations (28) and (29).

(28) $$H(X_{i}(t+1),0)=H(X_{i}(t),0)-\mathrm{randi}(Ub^{b}-H(X_{i}(t),0))+\mathrm{randi}(H(X_{i}(t),0)-Lb^{b})$$

(29) $$\left\{\begin{array}{l} Lb^{b}=\max(H(X^{b},0)-\frac{Dim}{2},0)\\ Ub^{b}=\min(H(X^{b},0)+\frac{Dim}{2},Dim) \end{array}\right.$$

In the above equation, $X^{b}$ represents the global optimal individual. The position update of the binary code string described by Equation (28) includes two steps. 1. Randomly select $\mathrm{randi}(Ub^{b}-H(X_{i}(t),0))$ code loci from all the loci with the value of “1” and $\mathrm{randi}(H(X_{i}(t),0)-Lb^{b})$ code loci from all the loci with the value of “0” in the current individual $X_{i}(t)$ to flip. If the number to flip exceeds the limit during each of the two flipping operations, flip all codes. 2. Check whether the Hamming distance of $X_{i}(t+1)$ after these two flipping operations exceeds the range specified by Equation (29) and perform a minimum number of random flips to guarantee that the boundary is not exceeded.

(4) Stealing dung beetles

The position update mechanism of the stealing individuals’ binary code strings is shown in Equation (30).

(30) $$H(X_{i}(t+1),X^{b})=\mathrm{randi}(\frac{H(X_{i}(t),X^{*})+H(X_{i}(t),X^{b})}{2})$$

According to Equation (30), when updating a stealing dung beetle individual, $\left[\frac{H\left(X_{i}\left(t\right),X^{*}\right)+H\left(X_{i}\left(t\right),X^{b}\right)}{2}\right]$ loci are randomly selected from all the loci of the global optimal individual $X^{b}$ to flip.

The individual’s update process is shown in the pseudocode of Algorithm 1.
**Algorithm 1:** The individual’s position update procedure.  **Input:** order quantity N, population size $Size_{p}$, current population ${pop}_{c}$.  **Output:** updated population ${pop}_{s}$.  1. initiate t=1  2. **While** $t<Size_{g}$ **do**  3.     $x_{t}^{c}={pop}_{c}(t,1)$  4.     $y_{t}^{c}={pop}_{c}(t,2)$  5.    **if**
t∈ ball-rolling subpopulation  6.     **if**
rand<0.9  7.      $x_{t}^{s}=Equation\left(18\right)(x_{t}^{c})$  8.      $y_{t}^{s}=Equation\left(25\right)(y_{t}^{c})$  9.     **else**  10.    $x_{t}^{s}=Equation\left(28\right)(x_{t}^{c})$  11.    $y_{t}^{s}=Equation\left(25\right)(y_{t}^{c})$  12.   **end if**  13.  **else if**
t∈ breeding subpopulation  14.    $x_{t}^{s}=Equation\left(20\right)(x_{t}^{c})$  15.    $y_{t}^{s}=Equation\left(26\right)(y_{t}^{c})$  16.  **else if**
t∈ small dung beetle subpopulation  17.    $x_{t}^{s}=Equation\left(23\right)(x_{t}^{c})$  18.    $y_{t}^{s}=Equation\left(28\right)(y_{t}^{c})$  19.  **else if**
t∈ stealing subpopulation  20.    $x_{t}^{s}=Equation\left(24\right)(x_{t}^{c})$  21.    $y_{t}^{s}=Equation\left(30\right)(y_{t}^{c})$  22.  **end if**  23.  ${pop}_{s}\left(t\right)=(x_{t}^{c},y_{t}^{c})$  24.  t=t+1  25. **end while****  Return**$\;{pop}_{s}$

#### 4.2.2. Hybrid Initialization Strategy

The global search ability of the swarm intelligence algorithm depends on the diversity of the initial population, which cannot be guaranteed by the random initialization process in the basic DBO. In order to improve the quality of the initial population, this paper proposes a hybrid initialization method of the chaotic map and a heuristic rule.

Circle mapping [45] is a kind of chaotic map with a good uniform distribution on [0,1]. In this paper, the chaotic map method shown in Equation (31) is used to generate the first-layer codes of most initial individuals, where $x_{i}(t)$ represents the i-th code value of the t-th individual. The second-layer codes of these initial individuals are generated by random initialization.

(31) $$x_{i}(t+1)=Lb+(Ub-Lb)\mathrm{mod}(\frac{x_{i}(t)-Lb}{Ub-Lb}+0.2-(\frac{0.5}{2\pi })\sin(2\pi x_{i}),1)$$

Because each task point has different adjacency distances from the left I/O point and the right I/O point, and the closer it is to the I/O point, the more obvious the difference is, we have reason to speculate that the optimal solution should have some characteristic of spatial delimitation. That is, the orders closer to the left tend to be assigned to the left stacker, while the orders on the right tend to be assigned to the right stacker. Without considering other optimization objectives, this characteristic of spatial delimitation not only meets the requirement of minimum operation times but also reduces the risk of stacker collisions to a certain extent.

According to the above ideas, in addition to the individuals generated by chaotic mapping, we also use a heuristic method to generate a small number of individuals to guide the iterative direction of the IDBO. The hybrid population initialization process is shown in the pseudocode of Algorithm 2.
**Algorithm 2:** The population initialization operator.  **Input:** problem instance, problem parameters (order quantity N, population size $Size_{p}$, variable range [Ub,Lb]).  **Output:** initial population.  1. Calculate the Chebyshev adjacency matrix between all points in the instance  2. Initialize the task sequences, SL=[], RL=[], t=0
  3. $Temp=circlemap(Size_{p},Ub,Lb,N)$  4. **While** $t<Size_{g}$ **do**  5.    **if** rand<0.9  6.     $x_{t}=Temp(t)$, $y_{t}=randi(2,[1,N])-1$  7.    **else**  8.     n=N
  9.     **while** n≥2 **do**  10.     $\left[O_{1},O_{2}\right]=$ Randomly select from the remaining order pool  11.    n=n−2
  12.    ${TS}_{1}=\{\left[SL,O_{1},O_{2}\right],RL\}$, ${TS}_{2}=\{\left[SL,O_{2},O_{1}\right],RL\}$, ${TS}_{3}=\{\left[SL,O_{1}\right],[RL,O_{2}]\}$
  13.    ${TS}_{4}=\{\left[SL,O_{2}\right],[RL,O_{1}]\}$, ${TS}_{5}=\{SL,[RL,O_{1},O_{2}]\}$, ${TS}_{6}=\{SL,[RL,O_{2},O_{1}]\}$
  14.    **for** i=1:6  15.     $t_{i}=ParallelOperateTime({TS}_{i})$  16.    **end for**  17.    $\left[\sim ,index]=min([t_{i}]\right)$  18.    $SL={TS}_{index}(1)$, $SR={TS}_{index}(2)$  19.   **end while**  20.   **if** n==1  21.    $\left[\sim ,index]=min([OperationTime\left(SL\right),OperationTime\left(RL\right)]\right)$  22.    **if** index==1  23.     $SL=[SL,O_{remain}]$  24.    **else** $RL=[RL,O_{remain}]$  25.    **end if**  26.   **end if**  27.   transcode {SL,RL} to the double-layer code individual $\left(x_{j},y_{j}\right)$
  28.  **end if**  29. **end while**  **Return** initial population $\left\{(x_{1},y_{1}),(x_{2},y_{2}),\cdots ,(x_{Size_{p}},y_{Size_{p}}),\right\}$

In Algorithm 2, the function ParallelOperateTime represents the process of calculating the parallel operating time based on the task sequence of the left and right stacker cranes. The specific calculation process refers to Steps 2–5 in Section 4.2.2. Unlike the complete decoding process, the input of the ParallelOperateTime function is the stacker crane task sequence, and only the fitness function F1 needs to be output. The function OperationTime represents the individual operating time of the left and right stacker cranes obtained during the calculation of the parallel operating time.

#### 4.2.3. Cauchy–Gaussian Mixture Distribution Neighborhood Search Operator

In order to avoid the algorithm prematurely falling into local optimization, this paper designs a neighborhood generation and reservation operator based on the Cauchy–Gaussian mixture distribution.

For a specified individual, the first-layer codes of its neighborhood individual are obtained after mutation, as in Equation (32).

(32) $$x_{i,d}^{new}(t)=x_{i,d}(t)+\frac{1-2^{-\gamma }}{3}(\beta_{1}\mathrm{Cauchy}(0,1)+\beta_{2}\mathrm{Gauss}(0,1))$$

Cauchy⁡(0,1) represents the standard Cauchy random number and Gauss⁡(0,1) represents the standard Gaussian random number. γ is the neighborhood magnification factor, taking $\gamma =\frac{Ub-Lb}{a_{d}-b_{d}}$, where $a_{d}$ is the upper bound of values in dimension d and $b_{d}$ represents the lower bound. $\beta_{1}$ and $\beta_{2}$ are parameters that represent the influence ratio of the two distributions. In this paper, take $\beta_{1}=1-\frac{t^{2}}{T^{2}},\beta_{2}=\frac{t^{2}}{T^{2}}$. This allows the Cauchy distribution to play a major role in the early stages of the iteration, and as the algorithm iterates, the influence of the Cauchy distribution will gradually decrease, while the influence of the Gaussian distribution will gradually increase and eventually replace the Cauchy distribution. Due to the probability distribution characteristics of the Cauchy distribution and Gaussian distribution, this hybrid mutation strategy can provide a larger mutation range at the beginning of the algorithm iteration and a more concentrated mutation range at the end of the algorithm. A wider mutation range in the early stage of the iteration helps to enhance the algorithm’s global search capability, while a slightly narrower mutation range in the later stage can concentrate this search activity within a local range, thereby improving the algorithm’s local search capability and avoiding excessive mutations that may affect convergence.

The second-layer code string of the neighborhood individual is generated by the traditional mutation method, that is, randomly selecting a code locus to flip its value to the reverse one, such as “flip 1 to 0” or “flip 0 to 1”.

In particular, for the optimal individuals of each generation, use reverse learning Equation (33) to generate two reverse individuals and then flip all the second-layer codes to obtain their neighborhood individuals.

(33) $$\left\{\begin{array}{l} x_{i}^{new}(1)=Ub+b_{i,d}-x_{i}\\ x_{i}^{new}(2)=Lb+a_{i,d}-x_{i} \end{array}\right.$$

$x_{i}^{new}\left(1\right)$ represents the values of the first reverse individual’s first-layer codes in the i-th iteration, $x_{i}^{new}\left(2\right)$ represents the values of the second reverse individual’s first-layer codes in the i-th iteration, Ub represents the upper boundary of the variable definition field, while Lb represents the lower boundary of the variable definition field, $b_{i,d}$ represents the minimum value in d-th dimension of the current population, while $a_{i,d}$ represents the maximum value in d-th dimension of the current population, and $x_{i}$ represents the values of the optimal individual’s first-layer codes in the i-th iteration.

In addition to the neighborhood individual generation approach, we provide the corresponding neighborhood evaluation criteria as follows:

If we use $X_{i}^{nei}(t)=\mathrm{neighbor}(X_{i}(t))$ to represent the generation of the neighborhood of individual $X_{i}(t)$ and $X_{i}(t+1)=\mathrm{step}(X_{i}(t))$ to represent the update of $X_{i}(t)$, then the neighborhood to be evaluated can be represented as $\left\{\mathrm{step}(X_{i}(t)),\mathrm{neighbor}(X(t+1)),\mathrm{step}(X_{i}^{nei}(t))\right\}$, and the individual with the best fitness value will be selected for the next generation population. The neighborhood population generation process is shown in the pseudocode of Algorithm 3.
**Algorithm 3:** The neighborhood population generation procedure.  **Input:** order quantity N, population size $Size_{p}$, current population ${pop}_{c}$.  **Output:** neighborhood population ${pop}_{n}$, optimal individual index.  1. Calculate the range of current population’s first-layer values, [a;b]  2. Initiate t=1  3. $\left[\sim ,index]=min(decode({pop}_{c})\right)$, retrieve the optimal individual index  4. **While** $t<Size_{g}$ **do**  5.    $x_{t}^{c}={pop}_{c}(t,1)$  6.    $y_{t}^{c}={pop}_{c}(t,2)$  7.    $x_{t}^{n}=Equation\left(32\right)(x_{t}^{c})$  8.    $y_{t}^{n}=randflip(y_{t}^{c})$  9.    ${pop}_{n}\left(t\right)=(x_{t}^{n},y_{t}^{n})$  10.  t=t+1  11. **end while**  12. $x_{N+1}^{n},x_{N+2}^{n}=Equation\left(33\right)(x_{index}^{c})$  13. $y_{N+1}^{n},y_{N+2}^{n}=randflip(y_{index}^{c})$  14. ${pop}_{n}\left(N+1\right)=(x_{N+1}^{n},y_{N+1}^{n})$, ${pop}_{n}\left(N+2\right)=(x_{N+2}^{n},y_{N+2}^{n})$
**Return** $\;{pop}_{n}$, index

#### 4.2.4. Velocity Revising Strategy Based on Continuous Space Discretization

When using metaheuristic algorithms such as the basic DBO to solve combinatorial optimization problems, there will be a problem called optimization distortion.

Without considering the performance of the algorithm, most combinatorial optimization problems can be solved directly using metaheuristic algorithms by relaxing integer variables into continuous variables. In this process, it is necessary to sort the continuous variables to transform them into a combinatorial optimization scheme. This sorting operation can be decomposed into pairwise comparisons between variables of different dimensions. The interface of size sorting between dimensions i and j can be represented as a hyperplane $x_{i}-x_{j}=0$, which we can refer to as the cutting plane. All cutting planes divide the original solution space into several subspaces, and all points in the same subspace correspond to the same discrete solution.

**Figure 10:**
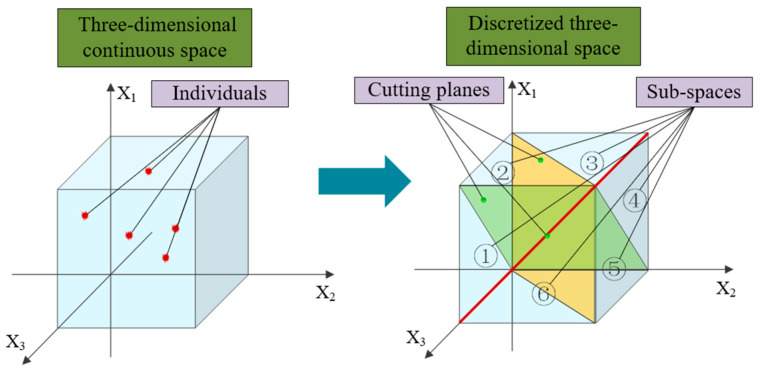
An example of continuous space discretization for the three-dimensional problem.

**Figure 11:**
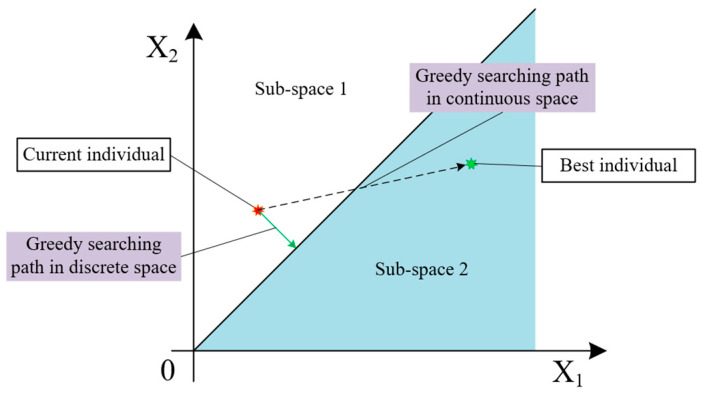
A two-dimensional example for the optimization distortion problem.

Figure 10 shows a simple and intuitive example of discretization in a three-dimensional continuous space, which can introduce the optimization distortion problem mentioned earlier. It can be clearly seen from the simplest two-dimensional example shown in Figure 11 that under the same optimization strategy, the direction and step size of optimization in a continuous space are significantly different from the actual situation considering spatial discretization, which may affect the speed and quality of algorithm optimization.

**Figure 12:**
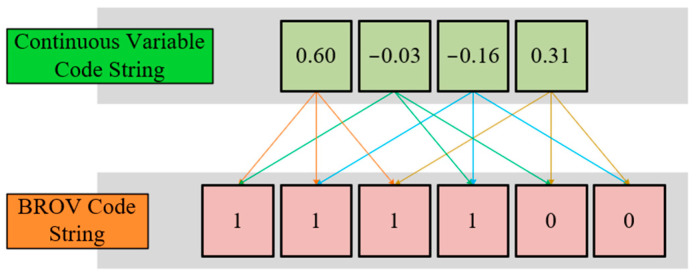
An example of the BROV generation process of a four-dimensional individual.

In order to compensate for the adverse effects caused by optimization distortion, this paper proposes an individual velocity revising strategy based on binary rank of value (BROV) encoding. Figure 12 shows how to generate a BROV code string for a four-dimensional individual, where each BROV code represents the positional relationship of the individual relative to a cutting plane. The D-value obtained by counterpoint subtracting an individual’s BROV code string from another can reflect their positional relationship relative to each cutting plane.

**Figure 13:**
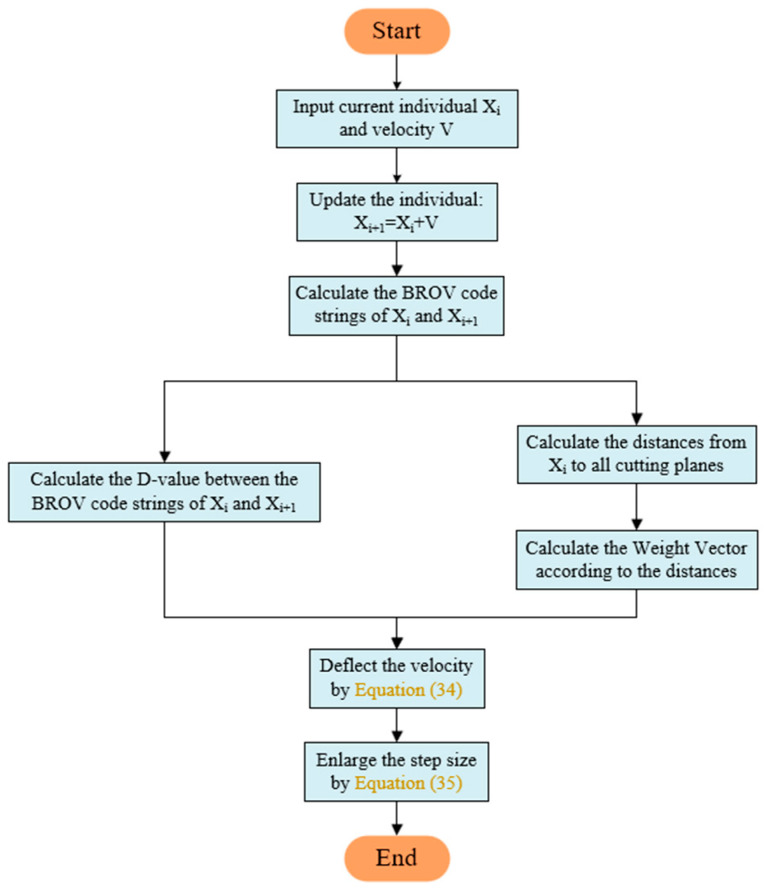
Velocity revising process based on continuous space discretization.

Based on this, we provide the individual’s position update revising process in Figure 13.

In the velocity revising process, direction revising refers to accumulating the obtained deflection direction with the original velocity direction according to Equation (34). The step size is adjusted according to Equation (35), which means that the minimum distance from the individual to all the cutting planes is used as the lower limit to adjust the step size of the normal component of the current individual’s velocity direction in that plane, ensuring penetrating at least one cutting plane after a position update.

(34) $$v_{d}=\frac{\left|v\right|}{\left|N\cdot {\left(\Delta b*d\right)}^{T}\right|}N\cdot {\left(\Delta b*d\right)}^{T}+v$$

(35) $$v_{s}=\frac{v_{d}}{\min(\min(|n_{i}\cdot v^{T}|/d_{i},1))}$$

In the above equations, v represents the original velocity, $v_{d}$ represents the direction-revised velocity, $v_{s}$ represents the step size-revised velocity, $N=[n_{1},n_{1},\cdot \cdot \cdot ,n_{nb}]$ represents the matrix composed of the normal vectors of all the cut planes, Δb represents the D-value vector of two individuals’ BROV code strings, and $d=[d_{1},d_{2},\cdot \cdot \cdot ,d_{nb}]$ represents the vector composed of the distance from the individual to each cut plane.

In the basic DBO, the position update of individuals corresponding to Equation (18) has continuity, which means that the ball-rolling individuals attempt to move away from the global worst solution.

We apply the velocity revising strategy to Equation (18) according to the following steps: First, calculate the D-value between the current individual and the global worst individual and then, based on the distance from the individual to the cutting planes, the normal vectors of the cutting planes are inversely weighted and accumulated to obtain the direction of revising velocity. When the distance from the individual to the cutting plane equals l, the weight coefficient is taken as $w=2^{-l}$. Finally, adjust the step size for this step of the position update.

In addition, the position updates of subpopulations corresponding to Equations (20), (22) and (24) belong to a local search, and their positions mainly update in the established regions. Therefore, we only need to adjust the step sizes for the position updates of these subpopulations. It should be noted that the velocity revising strategy is only applicable to the continuous layer.

The velocity revising process is shown in the pseudocode of Algorithm 4.
**Algorithm 4:** The individual’s velocity revising procedure.  **Input:** individual’s current first-layer code string x, updated first-layer code string x’, normal vectors’ matrix of the cut planes in N-dimensional continuous space N.  **Output:** revised first-layer code string $x_{r}^{\prime }$.  1. Calculate the current velocity $v=x^{\prime }-x$  1. Calculate the BROV code string of x and x,BROV(x), $BROV(x^{\prime })$  2. $\Delta b=BROV\left(x\right).-BROV(x^{\prime })$, contrapuntal subtraction  3. Calculate the distances from individual’s current position to all cut planes d  3. **if** individual ∈ ball-rolling subpopulation  4.  Deflect the velocity by Equation (34)  2.  Enlarge the step size by Equation (35)  3. **else**  3.  Enlarge the step size by Equation (35)  3. **end if**
  3. Calculate the revised position $x_{r}^{\prime }$
  **Return**
$\;x_{r}^{\prime }$


#### 4.2.5. Other Minor Modifications to DBO

- Improved Dance Strategy of the Dung Beetle

The basic DBO introduces a position update strategy under small probability to increase the randomness of ball-rolling individuals’ position updates, as shown in Equation (19), which can be called the tangent dance strategy. Due to the properties of the tangent function, when using this strategy, there is a 1/4 probability in each dimension to maintain the velocity direction and increase the velocity size, which can easily lead to the problem of updated positions exceeding the boundary due to excessive step size.

To solve this problem, this paper proposes a sine–cosine dance strategy, as shown in Equation (36). The improved dance strategy can limit the step size while preserving the randomness of position updates.

(36) $$x_{i,d}(t+1)=\left\{\begin{array}{l} x_{i,d}(t)+r_{1}\sin(\theta )|x_{i,d}(t)-x_{i}(t-1)|+r_{2}\cos(\theta ),rand>0.5\\ x_{i,d}(t)+r_{1}\cos(\theta )|x_{i,d}(t)-x_{i}(t-1)|+r_{2}\sin(\theta ),rand\leq 0.5 \end{array}\right.$$

$x_{i,d}(t)$ represents the value of the d-th dimension of individual i in the t-th iteration, θ∈(0,2π) represents a random angle value, and parameters $r_{1}$ and $r_{2}$ represent the historical dependency ratio. In this paper, $r_{1}=2(1-\frac{t^{2}}{T^{2}})$ and $r_{2}=0.5\frac{t^{2}}{T^{2}}(Ub-Lb)$ are taken.

- Local Search Boundary Modification

In the basic DBO, the feasible regions for updating the position of breeding dung beetles and small dung beetles are shown in Equations (21) and (22). Obviously, the feasible regions set in this way will be greatly affected by the value of $X^{*}$ and $X^{b}$. Therefore, in the IDBO, modifications will be made to the boundaries of these two subpopulations’ feasible regions for updating. Equation (37) specifies the modified update boundary for the breeding individuals, while Equation (38) specifies the modified update boundary for the small dung beetles.

(37) $$\left\{\begin{array}{l} Lb^{*}=\max(X^{*}-R\frac{Ub-Lb}{2},Lb)\\ Ub^{*}=\min(X^{*}+R\frac{Ub-Lb}{2},Ub) \end{array}\right.$$

$Lb^{*}$ and $Ub^{*}$ represent the lower and upper bounds of the safe zone, Lb and Ub represent the lower and upper bounds of the variable definition field, and $X^{*}$ represents the local optimal solution. $R=1-t/T_{max}$ and $T_{max}$ represent the maximum number of iterations.

(38) $$\left\{\begin{array}{l} Lb^{b}=\max(X^{b}-R\frac{Ub-Lb}{2},Lb)\\ Ub^{b}=\min(X^{b}+R\frac{Ub-Lb}{2},Ub) \end{array}\right.$$

$Lb^{b}$ and $Ub^{b}$ represent the lower and upper bounds of the foraging region and $X^{b}$ represents the global optimal solution.

### 4.3. Algorithm Process of IDBO

**Figure 14:**
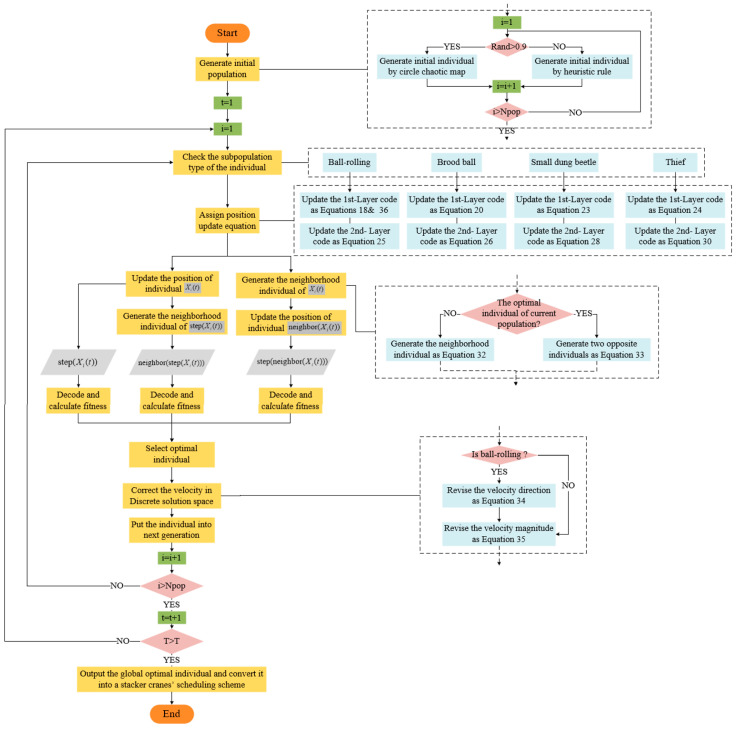
The flow chart of the IDBO for the TSSP.

After describing the key operators in detail, we give the flow chart of the IDBO for the TSSP, as shown in Figure 14.

## 5. Results and Discussion

This section will introduce the details of algorithm validation experiments and discuss the experimental results. Firstly, we set the problem parameters and introduce the experimental instances. Then, a set of experiments on the standard scale are conducted to discuss the sensitivity of algorithm parameters and determine the optimal parameters. Next, we conduct ablation experiments to discuss the performance of key improvement strategies in the IDBO and demonstrate that the IDBO can solve related problems and has certain performance advantages by solving a similar instance from the existing literature. Finally, the performance of the IDBO, the basic DBO, and other classical algorithms in solving TSSPs were investigated through several comparative experiments conducted on the instances with different-scale cases and the instances with different task distributions. All the algorithms in this section were programmed and solved by matlabR2022b software, and the experiments were run on a computer with an Intel (R) Core (TM) i7-12700 @ 2.10 GHz processer, 16G memory, and the Windows 11 operating system.

### 5.1. Experiment Settings

#### 5.1.1. Parameter Setting

This paper only discusses a high-rise shelf with 60 columns and 12 layers, which is common in shipyards. The specific problem parameters are shown in Table 2.

#### 5.1.2. Instance Generation

According to the average half-day throughput of the AS/RS in a shipyard in Southeast China, for a single-sided high-rise shelf, the total order quantity of 30 is taken as the standard problem scale. This paper generated five groups of instances of different scales for testing by setting N, the total number of orders, to 10, 20, 30, 40, 50, and 60. These instances were denoted as small-scale instances (C10, C20), the standard scale instance (C30), and large-scale instances (C40, C50, C60). Each instance is composed of a group of random orders, including information about location coordinates, superior pallet numbers, and order types. Each order generator includes two parameters, the total number of orders and the proportion of retrieved orders.

Specifically, we generated six additional groups of standard scale instances with different task distributions to enrich the comparative experiments. Within the instances, D30-1 is a uniformly distributed instance. D30-2 is an instance mainly distributed on the left side of the shelf, which was generated by a generator with the orders’ horizontal coordinates following X~Pois(λ=4). D30-3 is an instance where orders are concentrated in the middle of the shelf, generated by a generator with the orders’ horizontal coordinates following $X\sim N(30,15^{2})$. P30-1, P30-2, and P30-3 represent the instances where retrieval orders account for 20%, 50%, and 80% of the total orders, respectively.

For length reasons, we only give the specific information of C30, D30-series, and P30-series instances, as shown in Table 3.

### 5.2. Sensitivity Analysis of IDBO’s Parameters

**Figure 15:**
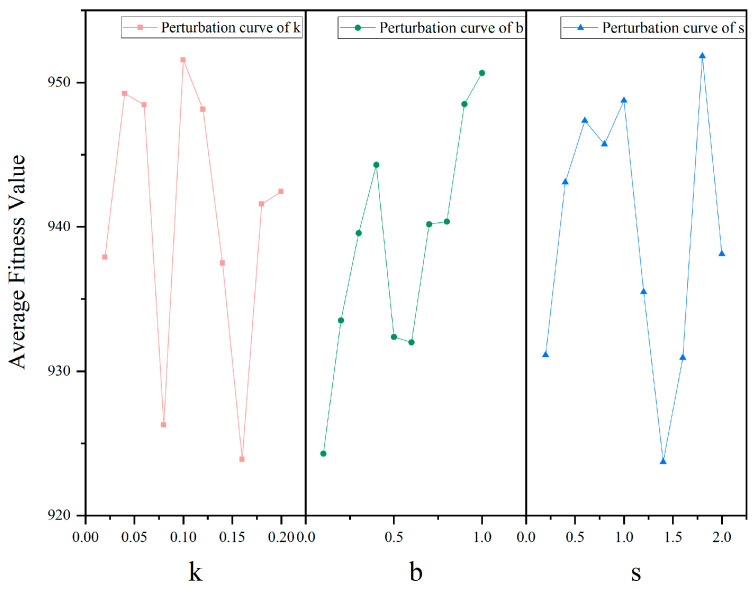
Disturbance curves of control parameters.

The IDBO employed three control parameters (k, b in Equation (18) and s in Equation (24)). In order to analyze the sensitivity of each parameter when solving the TSSP and determine the optimal parameter combination, we conducted ten disturbance experiments on each parameter. The study 30 has stipulated the value ranges of control parameters as $k\in \left(0,0.2\right],b\in (0,1],\;s\in (0,2]$ and recommended the values to be k=0.1,b=0.3,s=0.5. On this basis, the parameters’ values of disturbance experiments are given in Table 4. The experimental results are shown in Figure 15.

It is observable in Figure 15 that all parameters show a certain degree of robustness, as the fluctuation in the evaluation index (average fitness value, AFV) in all disturbance experiments has not exceeded 5%. The parameter k demonstrates the most sensitive behavior relatively. The sensitivities of all three parameters have a certain degree of multimodal characteristics; that is, there are some relatively more sensitive interval segments in the disturbance interval. For example, the sensitivity of s is significantly improved between interval segments [1,1.8]. The above experimental results demonstrate that the IDBO has good stability when the parameters are set within the recommended interval. However, the influence of parameters on the IDBO’s performance is complex. The algorithm parameters need to be carefully adjusted for specific problems to achieve the best performance.

After completing the disturbance experiments, select three values (which make the evaluation index take the minimum value, median value, and 80% interval value) of each parameter. Based on this, a total of 27 groups of full factor experiments were conducted with three factors and three levels; the factor level values for these three parameters are shown in Table 5. Each experiment was conducted 30 times, and the average results are recorded in Table 6. Therefore, we set the parameters of the IDBO as k=0.16,b=0.1,s=2.

### 5.3. Ablation Study

The IDBO’s improvement strategies include four key components, which can be called the initialization component (IC), neighborhood search component (NSC), double-layer coding component (DLCC), and velocity revising component (VRC). In order to understand the specific contribution of these key components to the overall performance of the IDBO, multiple ablation experiments were conducted in this paper. Each ablation experiment was performed 30 times and recorded with the average value. The maximum number of iterations is set to 500. The experimental results (average fitness value, AFV, and average convergence iterations, ACI) are recorded in Table 7.

From the results in Table 7, it is observable that when we remove the double-layer coding component and the neighborhood search component separately, the overall performance of the algorithm suffers significant degradation (except for C10, the average decrease was 8.43% and 27.56%), especially when removing the double-layer coding component (27.56%). It indicates that both the double-layer coding component and the neighborhood search component can significantly improve the accuracy of the IDBO when solving the TSSP, and the contribution of the double-layer coding component is particularly critical.

When the velocity revising component is removed, although the accuracy of the algorithm does not show significant degradation, the convergence speed decreases significantly (the average decrease was 41.5%), which demonstrates that the velocity revising component plays a key role in improving the convergence speed of the IDBO. When the initialization component is removed, the solution accuracy (the average decrease was 1.4%) and convergence speed (the average decrease was 8.9%) of the algorithm show a certain degree of degradation, which indicates that the introduction of the initialization component can improve the solution accuracy and convergence speed of the IDBO to a certain extent.

The performance of the double-layer coding component, which makes the most outstanding contribution to the performance of the IDBO, meets the expectation corresponding to the mathematical characteristics of the TSSP. In order to qualitatively analyze the characteristics of the TSSP and discuss the reasons for the performance contribution of the double-layer coding component, the TSSP is re-modeled according to the bi-level programming theory. First, we simplify the optimization goal to the minimum parallel makespan. Under this assumption, the first level of the TSSP bi-level programming model is the task grouping model, which takes the minimum parallel makespan as the optimization objective without considering collision. The upper planning model is shown as follows:

(39) $$\underset{x}{\min}F(x,y)$$

(40) $$s.t.\sum_{n}\sum_{m}x_{mn}=N$$

(41) $$\sum_{n}x_{mn}=1$$

where $x_{mn}$ is the decision variable. $x_{mn}=1$ when the task m is assigned to the stacker n, otherwise $x_{mn}=0$.

The lower-level planning is a task scheduling problem with a minimum parallel makespan, shown as follows:

(42) $$\underset{y}{\min}f(x,y)$$

(43) $$s.t.\sum_{j}\sum_{k}y_{ijk}\leq 1$$

(44) $$\sum_{i}\sum_{k}y_{ijk}\leq 1$$

(45) $$(y_{i1}+\sum_{j}y_{ij1})(y_{i2}+\sum_{j}y_{ij2})=0$$

(46) h(x,y)≤0

where $y_{ijk}$ is the decision variable. $y_{ijk}=1$ indicates that task i is ahead of j in the task sequence of stacker k.

The model constructed above is a typical optimistic bi-level programming model [46]. Based on the bi-level programming theory, the performance of the model solving algorithm depends on the search ability of the algorithm in the guidance domain [47].

Because the search ability in the guidance domain is equivalent to the synchronous search ability in the upper and lower constraint domains, when the bi-level programming problem adopts single-layer coding, the search ability of the algorithm in the upper and lower planning feasible region will show a non-equal state, especially for the TSSP, the upper planning scheme is only affected by the coding of the code with the minimum value in the coding string, so that the search ability of the algorithm will show a serious imbalance state, and the severity of this problem will increase exponentially with the expansion of the scale. Therefore, for multi-layer optimization problems, designing corresponding multi-layer coding is a necessary means to ensure the performance of the algorithm.

### 5.4. Instance Validation

In this part, we will compare the IDBO with the existing method for problems similar to the TSSP and give the final stackers’ scheduling scheme. The study 18 also discusses the collision problem of double stackers; we name the instance in study 18 “G-instance”. The G-instance and the optimal result are shown in Table 8. In the process of instance validation, we revised the objective function to the minimum parallel operation time to be consistent with study 18.

**Figure 16:**
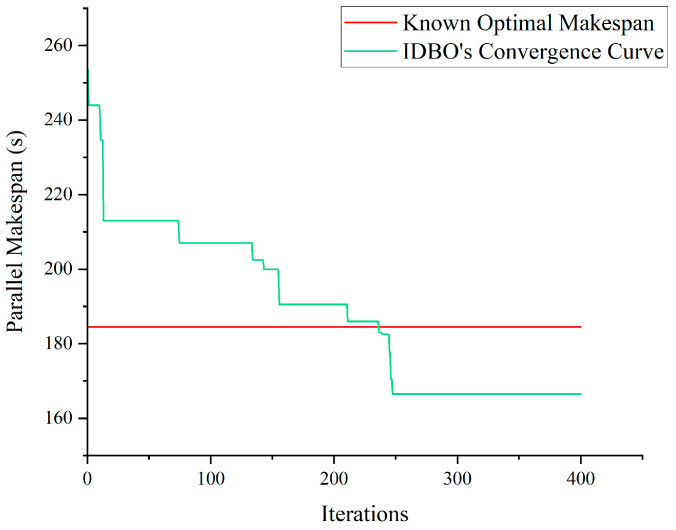
The convergence curve of the IDBO for solving the G-instance.

Figure 16 shows the convergence curve corresponding to this instance. It is observable that the IDBO has reached the optimal solution given by study 18 in generation 236, and the optimal makespan obtained by the IDBO is 10% less than that given by study 18.

**Figure 17:**
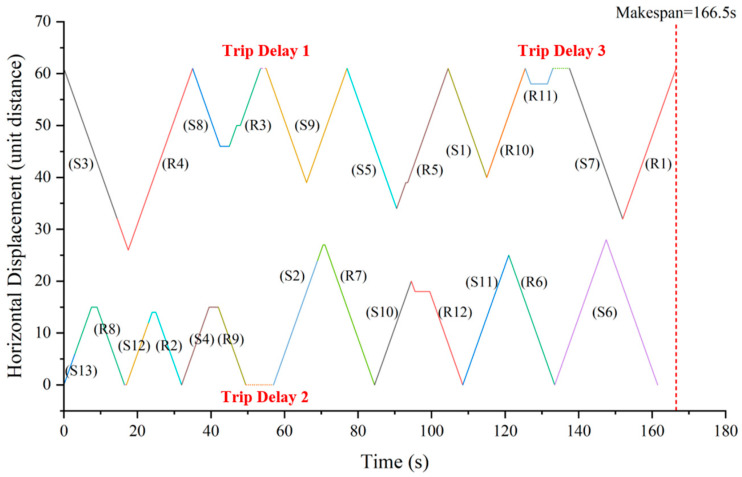
The x-t trajectory of the two stackers corresponding to the optimal solution.

Table 9 presents the stackers’ scheduling scheme corresponding to the solution obtained by the IDBO. Figure 17 shows the x-t trajectory of the two stackers corresponding to the scheduling scheme. It is easy to verify that the scheduling scheme meets all constraints of the TSSP.

### 5.5. IDBO’s Performance on Various-Scale Instances

This subsection mainly discusses the performance of the IDBO algorithm and other classical algorithms when solving the TSSP with various scales. In order to select the suitable comparison algorithms, we conducted simple TSSP instance solving tests on over 60 well-studied metaheuristic algorithms in recent years and selected five algorithms with the best performance, namely the gray wolf optimizer (GWO) [48], the whale optimization algorithm (WOA) [49], the white shark optimizer (WSO) [50], the elk herd optimizer (EHO) [51], and the covariance matrix adaptation evolution strategy (CMA-ES) [52], as well as the most classical population-based algorithm, particle swarm optimization (PSO) [53]. In addition, due to the mature continuous and discrete forms of genetic algorithms (GAs) [54], we can directly introduce the double-layer mechanism into a GA.

To ensure fairness, the experiments were conducted with the same population size (N=80), the same maximum iteration number ($T_{max}=300$) and the same termination criteria (reaching the maximum number of iterations), and the same performance metrics (average fitness value calculated in Equation (3)). The specific parameter settings for each algorithm are shown in Table 10.

In order to make the comparison results more rigorous, the Wilcoxon signed-rank test [55] is employed at the significance level of 5% confidence to verify whether the IDBO algorithm has significant performance advantages over other classic algorithms.

Table 11 records the average fitness value (AFV) and computing time (CT) of the IDBO and other tested algorithms after solving instances C10~C60 30 times, as well as the *p*-value of the Wilcoxon signed-rank test. In the last row of Table 11, the symbol “+” indicates that the IDBO is significantly superior to other results.

After excluding the maximum and minimum gap values, the average performance advantages of the IDBO over other algorithms are as follows: 20% better than the DBO, 9.4% better than the GWO, 21.2% better than the GA, 19% better than PSO, 16% better than the WOA, 22.1% better than the WSO, 19.5% better than the EHO and 20% better than the CMA-ES. From the trend in performance advantage, the IDBO has the most significant performance advantage on the 40-scale instance, and this performance advantage does not significantly decrease with the expansion of the instance scale. Therefore, it is observable that the performance of the IDBO is significantly better than other classical algorithms within the scope of the scales covered by this test.

When only considering the influence of the problem size N on the algorithms’ time complexity, the compared algorithms’ time complexity is O(N). The time complexity of the IDBO is $O(N^{2})$, since the velocity revising component needs to calculate the BROV code strings of all individuals in each iteration. From the computing time of each algorithm recorded in Table 11, it can be seen that the IDBO needs to pay more calculation time costs than the compared algorithms for the same-scale instance, and this calculation time cost will become increasingly prominent with the expansion of the problem scale. However, the computing time of the IDBO is completely acceptable within the general workload of the AS/RS in the shipyard.

**Figure 18:**
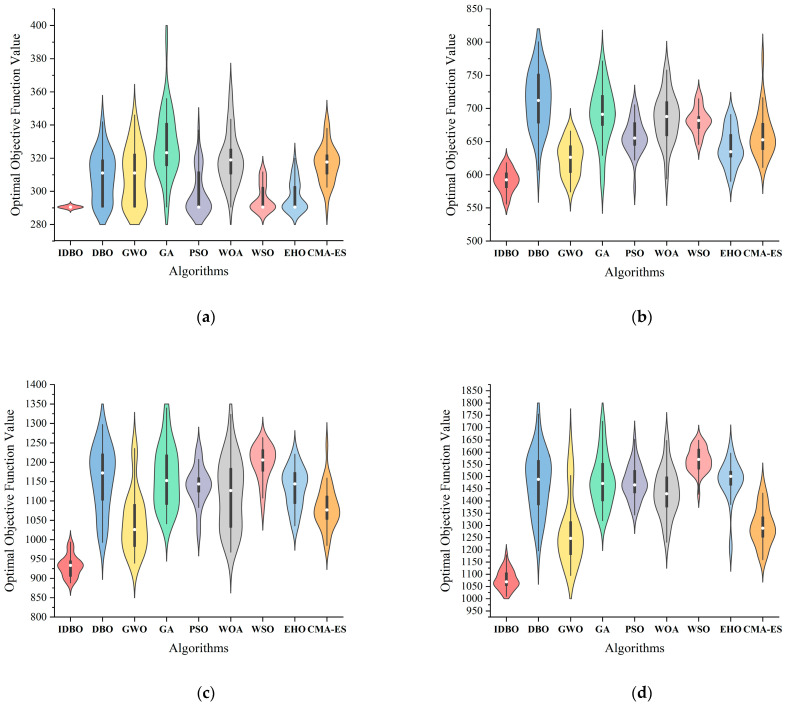

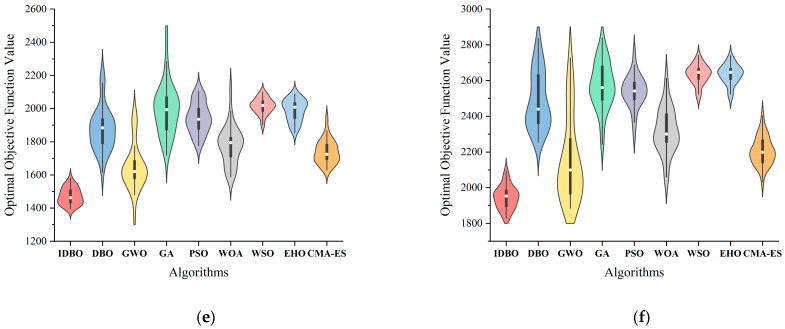
The violin plots of the optimal results obtained by the IDBO and other comparative algorithms on the test instances. (**a**) Results on instance C10; (**b**) results on instance C20; (**c**) results on instance C30; (**d**) results on instance C40; (**e**) results on instance C50; (**f**) results on instance C60.

In the form of violin plots, Figure 18 shows the solution results of the compared algorithms on various-scale instances. From Figure 18a–f, it is observable that the IDBO has better exploration ability and convergence than other compared algorithms and can effectively avoid falling into local optimization. This significant performance advantage will gradually increase with the expansion of the problem scale. However, it cannot be avoided that the results of the IDBO for large-scale instances have a certain divergence, which indicates that the convergence performance of the algorithm still has room for further optimization.

### 5.6. IDBO’s Performance on Various-Distribution Instances

Considering the significant impact of the instance’s task distribution on the value of the objective function, we added additional comparative experiments on standard-scale instances with different task distributions to verify whether the superior performance of the IDBO in solving TSSPs is general and realistic.

**Figure 19:**
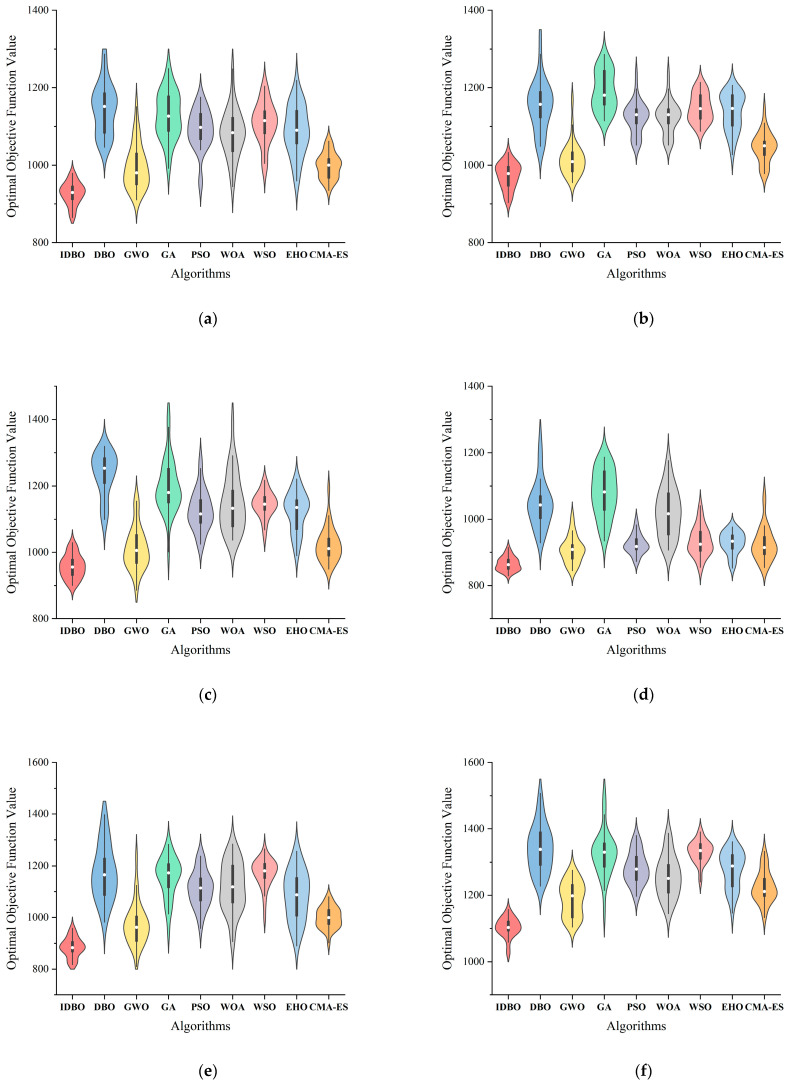
The violin plots of the optimal results obtained by the IDBO and other comparative algorithms on the various-distribution instances. (**a**) Results on instance D30-1; (**b**) results on instance D30-2; (**c**) results on instance D30-3; (**d**) results on instance P30-1; (**e**) results on instance P30-2; (**f**) results on instance P30-3.

Table 12 records the average results of the IDBO and other tested algorithms after solving various-distribution instances (C30, D30-1, D30-2, D30-3, P30-1, P30-2, P30-3) for 30 times, as well as the *p*-value of the Wilcoxon signed-rank test. Figure 19 shows the optimal solution results obtained by the IDBO and other comparative algorithms on these instances in the form of violin plots.

The experimental results in Figure 19 show that the IDBO’s performance is more competitive than other compared algorithms under the various task distributions tested; the average performance advantages of the IDBO over other algorithms are as follows: 19.5% better than the DBO, 6.25% better than the GWO, 19.4% better than the GA, 14% better than PSO, 15.5% better than the WOA, 16.13% better than the WSO, 13.8% better than the EHO and 8.07% better than the CMA-ES. It is worth noting that the IDBO maintains a stable and centralized result distribution in all experiments, which confirms that the IDBO has a reliable and universal ability to solve TSSPs.

## 6. Managerial Implications

This paper aims to solve the TSSP during the storage and retrieval stages of a large AS/RS in shipyards so as to provide an optimal scheduling scheme with a collision-free constraint. The current instruction input operation mode of stacker cranes in shipyards usually uses real-time avoidance or setting priority rules to handle stacker cranes’ collisions. This mode of dealing with collisions after they occur seriously affects the overall operational efficiency of the AS/RS. In addition, after being retrieved by stacker cranes, the materials in the AS/RS need to go through pallet picking operations before being distributed. When scheduling stacker cranes, not only the overall operation time but also the picking time of each pallet need to be considered. However, existing collision-free scheduling methods lack performance advantages when considering this comprehensive indicator as the optimal objective. The relaxed trip trajectories proposed in this paper transform the difficult problem of directly finding optimal collision-free solutions in complex solution spaces into a conventional problem of finding optimal solutions in sub-solution spaces that are subject to the collision-free constraint, thereby transforming the TSSP into a conventional problem which can be solved by heuristic optimizers.

In response to the TSSPs in shipyard environments, this paper proposed a superior IDBO compared with other metaheuristic algorithms. The collision-free scheduling scheme obtained by the IDBO ensures operational safety while also considering energy consumption and efficiency. By adjusting the value of α and β in Equation (3), the enterprise managers can adjust the optimization tendency of the IDBO in solving TSSPs (i.e., focusing more on optimizing the average preparation time of pallets by increasing α while focusing more on optimizing additional transportation energy consumption by increasing β). Furthermore, due to the stability of the twin stacker system structure and the excellent performance of the IDBO in solving small-scale instances, the IDBO has a certain dynamic scheduling ability. When one stacker fails, the TSSP can easily degenerate to a single stacker scheduling problem by fixing the second-layer codes to the residual stacker. When an emergency temporary order suddenly appears, it can be rescheduled in the time slot affected by the order to deal with the emergency. In summary, the IDBO potentially contributes to breaking through the operational efficiency bottleneck of large AS/RSs in shipyards, which is crucial for supporting the development of shipyard shipbuilding capabilities.

## 7. Conclusions

In order to solve the collision problem in the process of the twin stacker cranes’ storage and retrieval task scheduling in the shipyard’s large AS/RS, this paper proposes a trip trajectory relaxation method to simplify the collision identification and resolution process and formulates a mathematical model of the TSSP on this basis. Furthermore, this paper introduces a metaheuristic algorithm DBO to solve the large-scale TSSP and make special improvements to the basic DBO according to the characteristics of TSSPs. The IDBO contains improvement components such as the double-layer code mechanism, the hybrid initialization strategy, the Cauchy–Gaussian mixture neighborhood search strategy, and the velocity revising strategy based on continuous space discretization. In particular, according to the design idea of the DBO, the binary position update equations are proposed for the binary coding string of the double-layer code structure. In addition, the IDBO also revises the dung beetle dance strategy and the local search boundary, which are not applicable to the TSSP.

The performance advantages of the IDBO have been confirmed by a series of experiments. The ablation study demonstrates that the four improved components make outstanding contributions to the performance improvement of the IDBO. The instance validation shows that the IDBO can provide a competitive optimal scheduling scheme when solving problems similar to the TSSP in the existing literature. Through several groups of comparative experiments on different-scale and different-distribution instances, it has been proved that the IDBO has significant performance advantages over several well-studied classical algorithms (DBO, GWO, GA, PSO, WOA, WSO, EHO, and CMA-ES) in solving TSSPs. This advantage also includes the convergence stability of the IDBO on various instances, which means that the IDBO is expected to become a reliable and universal TSSP solving algorithm that can be used in the actual working environment of the AS/RS in the shipyard.

The IDBO proposed in this paper can provide a more efficient scheduling scheme for twin stacker crane units, but there are certain limitations to our study. Firstly, this paper assumes two limiting conditions; one is that the stacker crane has a single shuttle and the other is static scheduling. These two reasonable assumptions proposed under the current shipyard management mode may be overturned with the development of shipbuilding models. However, the IDBO cannot yet solve the problem of the dynamic scheduling of multiple multi-shuttle stacker cranes. Another limitation is that the IDBO has a higher time complexity than other metaheuristic algorithms, which limits its efficiency in solving larger scale TSSPs.

Therefore, for future work, we intend to expand this study as follows: 1. add dynamic orders into the order pool and consider the dynamic TSSP; 2. expand the single-shuttle stacker to the multiple-shuttle stacker; and 3.optimize the framework and components of the IDBO to enhance computational efficiency and given the competitive potentiality of the GWO and CMA-ES in experiments, developing algorithms based on these two frameworks would be a valuable pursuit. These three research directions are full of challenges and have profound engineering application significance.

## Figures and Tables

**Table 1:** Variables, parameters, and their definition.

Notation	Definition
$J_{1}$	Task point set of storage orders
$J_{2}$	Task point set of retrieval orders
P	Set of retrieval orders belonging to pallet P $,\;\mathrm{with}\;J_{2}=\{P_{1},P_{2},\cdot \cdot \cdot ,P_{N}\}$
$J=J_{1}\cup J_{2}$	Entire order pool contains all task points
$D=\{D_{1},D_{1}^{\prime },D_{2},D_{2}^{\prime }\}$	I/O point set, contains left and right I/O points and mirror points as the end of the trip
V=J∪D	Point set containing all order points and I/O points
$K=K^{L}\cup K^{R}$	Trip set of the current order pool, where $K^{L}=\{K_{s}^{L}\},s=1,2,\cdot \cdot \cdot ,|J|$ represents left stacker trips and $K^{R}=\{K_{s}^{R}\},s=1,2,\cdot \cdot \cdot ,|J|$ represents right stacker trips
$D_{k}$	I/O point corresponding to trip k
$L^{t}$	Total time of stacker to go through shelf in horizontal direction
$t_{ij}$	Travel time from point i to point j, calculated by Chebyshev distance
$t_{ij}^{o}$	Operation time of arc (i,j) , equals 0 if i,j belongs to D $,\;\mathrm{else}\;\mathrm{equals}\;a\;\mathrm{fixed}\;\mathrm{value}\;t_{o}$
$x_{ijk}\in \{0,1\}$	Decision variable: equals 1 if j subsequent to i in stacker’s task list of trip k, else equals 0
$s_{ik}\in R$	Decision variable: time when stacker leaves task point i in trip k

**Table 2:** Problem parameter specification.

Parameter	Value
Coordinate of left I/O point	(0, 1)
Coordinate of right I/O point	(61, 1)
Stacker’s horizontal speed	1 column/s
Stacker’s vertical speed	0.4 layers/s
Time of single loading/unloading operation	5 s

**Table 3:** Specific information of some instances.

Instance Name	Storage Order Content	Retrieval Order Content
C30	(29, 9) ^1^ (53, 4) (55, 8) (16, 4) (8, 4) (28, 3) (17, 5) (27, 6) (33, 1) (51, 6) (1, 9) (5, 3) (45, 2)	(35, 6, 4) ^2^ (13, 2, 4) (9, 8, 4) (48, 1, 1) (31, 8, 2) (16, 10, 2) (18, 2, 1) (27, 11, 4) (32, 6, 2) (4, 11, 5) (13, 7, 4) (52, 3, 5) (23, 5, 5) (43, 2, 6) (48, 6, 5) (22, 5, 1) (36, 7, 1)
D30-1	(49, 11) (38, 2) (58, 12) (17, 1) (12, 6) (43, 10) (40, 2) (23, 7) (35, 6) (10, 10) (10, 8) (42, 9) (14, 11) (33, 12)	(58, 6, 1) (26, 11, 6) (40, 1, 6) (41, 10, 3) (40, 3, 1) (42, 4, 1) (27, 5, 5) (58, 5, 2) (46, 4, 5) (54, 12, 1) (9, 4, 2) (49, 3, 3) (12, 4, 3) (22, 10, 4) (56, 4, 5) (32, 10, 1)
D30-2	(19, 2) (26, 3) (2, 5) (42, 7) (25, 3) (3, 1) (18, 5) (13, 4) (11, 4) (7, 8) (34, 11) (40, 10) (34, 10) (11, 8) (27, 1)	(5, 12, 5) (40, 11, 1) (11, 11, 6) (55, 6, 4) (13, 9, 3) (34, 2, 5) (33, 5, 6) (27, 5, 6) (25, 7, 3) (40, 8, 4) (13, 7, 4) (33, 4, 1) (26, 8, 2) (59, 11, 6) (27, 5, 3)
D30-3	(13, 1) (29, 12) (31, 6) (4, 5) (3, 8) (34, 11) (52, 3) (39, 7) (33, 5) (30, 5) (57, 4) (21, 9) (43, 10) (25, 3) (17, 3)	(35, 10, 6) (27, 10, 3) (25, 12, 5) (79, 7, 3) (39, 5, 6) (49, 7, 5) (4, 5, 1) (47, 1, 1) (38, 12, 5) (47, 8, 5) (28, 4, 6) (48, 6, 1) (53, 2, 3) (13, 9, 5) (32, 10, 6)
P30-1	(12, 5) (10, 11) (12, 6) (36, 3) (16, 4) (50, 12) (50, 4) (26, 4) (42, 9) (5, 4) (25, 10) (32, 4) (10, 4) (39, 12) (18, 9) (16, 3) (21, 10) (28, 6) (20, 10) (11, 9) (21, 8) (15, 12) (12, 4) (42, 7)	(2, 7, 5) (36, 2, 6) (26, 2, 3) (47, 6, 2) (28, 11, 6) (37, 5, 1)
P30-2	(56, 9) (32, 3) (41, 5) (6, 4) (9, 9) (49, 7) (54, 1) (4, 9) (26, 10) (40, 8) (1, 12) (23, 3) (58, 12) (26, 12)	(6, 4, 1) (15, 6, 4) (3, 11, 5) (30, 10, 6) (54, 5, 2) (2, 9, 3) (55, 8, 6) (59, 9, 3) (32, 2, 5) (44, 2, 4) (59, 8, 3) (11, 5, 5) (4, 5, 3) (17, 6, 6) (40, 7, 4) (11, 2, 2)
P30-3	(14, 2) (32, 7) (42, 9) (50, 6) (23, 3) (12, 6)	(39, 9, 6) (13, 9, 1) (37, 6, 4) (47, 5, 3) (51, 10, 4) (35, 7, 2) (20, 2, 4) (29, 8, 4) (33, 9, 6) (25, 6, 5) (38, 10, 6) (12, 2, 1) (24, 9, 4) (21, 2, 2) (3, 10, 3) (42, 5, 3) (20, 6, 2) (47, 5, 5) (20, 9, 6) (47, 3, 6) (31, 11, 1) (12, 5, 5) (48, 4, 1) (7, 2, 3)

^1^ Coordinates of the storage order. ^2^ Abscissa, ordinate, and superior pallets’ numbers of the retrieval order.

**Table 4:** Parameter values of single-factor disturbance experiments.

NO.	k-Value Disturbance			b-Value Disturbance			s-Value Disturbance		
	k	b	s	k	b	s	k	b	s
1	0.02	0.3	0.5	0.1	0.1	0.5	0.1	0.3	0.2
2	0.04	0.3	0.5	0.1	0.2	0.5	0.1	0.3	0.4
3	0.06	0.3	0.5	0.1	0.3	0.5	0.1	0.3	0.6
4	0.08	0.3	0.5	0.1	0.4	0.5	0.1	0.3	0.8
5	0.1	0.3	0.5	0.1	0.5	0.5	0.1	0.3	1
6	0.12	0.3	0.5	0.1	0.6	0.5	0.1	0.3	1.2
7	0.14	0.3	0.5	0.1	0.7	0.5	0.1	0.3	1.4
8	0.16	0.3	0.5	0.1	0.8	0.5	0.1	0.3	1.6
9	0.18	0.3	0.5	0.1	0.9	0.5	0.1	0.3	1.8
10	0.2	0.3	0.5	0.1	1	0.5	0.1	0.3	2

**Table 5:** Experimental factor and level settings.

Level	Factors		
	k	b	s
1	0.16	0.1	1.4
2	0.18	0.3	2
3	0.06	0.4	0.6

**Table 6:** The results of full factorial experiments for parameter setting.

NO.	Parameter Value			AFV	No.	Parameter Value			AFV	No.	Parameter Value			AFV
	k	b	s			k	b	s			k	b	s	
1	0.16	0.1	1.4	927.95	10	0.18	0.1	1.4	915.98	19	0.06	0.1	1.4	932.07
2	0.16	0.1	2	911.36	11	0.18	0.1	2	934.35	20	0.06	0.1	2	934.33
3	0.16	0.1	0.6	942.22	12	0.18	0.1	0.6	926.15	21	0.06	0.1	0.6	933.84
4	0.16	0.3	1.4	942.88	13	0.18	0.3	1.4	931.77	22	0.06	0.3	1.4	947.6
5	0.16	0.3	2	925.2	14	0.18	0.3	2	938.73	23	0.06	0.3	2	940.77
6	0.16	0.3	0.6	941.01	15	0.18	0.3	0.6	935.61	24	0.06	0.3	0.6	943.62
7	0.16	0.4	1.4	942.52	16	0.18	0.4	1.4	941.58	25	0.06	0.4	1.4	953.43
8	0.16	0.4	2	940.72	17	0.18	0.4	2	934.87	26	0.06	0.4	2	951.38
9	0.16	0.4	0.6	949	18	0.18	0.4	0.6	942.8	27	0.06	0.4	0.6	946.2

**Table 7:** Ablation study of IDBO with different-scale instances.

	**C10**			**C20**			**C30**		
	**AFV**	**Gap**	**ACI**	**AFV**	**Gap**	**ACI**	**AFV**	**Gap**	**ACI**
IDBO	290.5	-	33	588.53	-	252	929.48	-	291
(w/o) IC	290.5	0.00%	32	595.68	1.21%	283	936.19	0.72%	312
(w/o) NSC	303.4	4.44%	38	642.49	9.17%	277	989.55	6.46%	285
(w/o) DLCC	292	0.52%	35	648.9	10.26%	265	1126.46	21.19%	- *
(w/o) VRC	290.5	0.00%	46	594.16	0.96%	341	927.06	−0.26%	397
basic DBO	314.78	8.36%	245	689.66	17.18%	277	1136.23	22.24%	- *
	**C40**			**C50**			**C60**		
	**AFV**	**Gap**	**ACI**	**AFV**	**Gap**	**ACI**	**AFV**	**Gap**	**ACI**
IDBO	1076.6	-	262	1476.84	-	283	1945.68	-	276
(w/o) IC	1094.87	1.70%	307	1497.39	1.39%	299	1982.45	1.89%	314
(w/o) NSC	1180.2	9.62%	279	1583.26	7.21%	303	2134.49	9.70%	282
(w/o) DLCC	1462.11	35.81%	- *	1959.9	32.71%	- *	2681.39	37.81%	- *
(w/o) VRC	1073.36	−0.30%	379	1490.82	0.95%	434	1954.44	0.45%	386
basic DBO	1489.22	38.33%	- *	1962.95	32.92%	- *	2488.28	27.89%	- *

* Prematurely fall into local optimum.

**Table 8:** Instance details and the optimal result in the literature [18].

Storage Orders	No.	S1	S2	S3	S4	S5	S6	S7
	Coordinates	(40, 6)	(24, 8)	(32, 7)	(15, 11)	(34, 4)	(28, 3)	(32, 12)
	No.	S8	S9	S10	S11	S12	S13	
	Coordinates	(46, 11)	(39, 10)	(20, 4)	(25, 2)	(12, 6)	(6, 3)	
Retrieval Orders	No.	R1	R2	R3	R4	R5	R6	R7
	Coordinates	(35, 11)	(14, 8)	(50, 8)	(26, 9)	(39, 7)	(9, 5)	(27, 6)
	No.	R8	R9	R10	R11	R12		
	Coordinates	(15, 9)	(5, 11)	(45, 4)	(58, 7)	(18, 9)		
Optimal Makespan (s)	184.5							

**Table 9:** The stackers’ scheduling scheme obtained by the IDBO.

Left Stacker’s Task Sequence	Trip No.	1	2	3	4	5	6	7
	Order	(S13/R8)	(S12/R2)	(S4/R9)	(S2/R7)	(S10/R12)	(S11/R6)	(S6)
Right Stacker’s Task Sequence	Trip No.	1	2	3	4	5	6	7
	Order	(S3/R4)	(S8/R3)	(S9)	(S5/R5)	(S1/R10)	(R11)	(S7/R1)

**Table 10:** Specific parameter settings for compared algorithms.

Algorithm	Parameter/Operator	Value/Description
IDBO	{k, b, s}	{0.16, 0.1, 2}
DBO	{k, b, s}	{0.1, 0.3, 0.5}
GWO	$\left\{a_{min},\;a_{max}\right\}$	{0, 2}
GA	$\left\{p_{c},\;p_{m}\right\}$	{0.8, 0.05}
	Encoding mechanism	Double layer
	Selection operator	Roulette wheel based on fitness
	Crossover operator	Single-point crossover
	Mutation operator	Single-point mutation
PSO	${\{c}_{1},c_{2}\}$	{2, 2}
	Inertia weight	Linear reduction from 0.9 to 0.1
	Topology	Fully connected
WOA	a	Linearly decreased from 2 to 0
WSO	$\left\{a_{0},a_{1},a_{2}\right\}$	{6.25,100,0.0005}
	$t_{au}$	4.11
	$\left\{f_{min},f_{max}\right\}$	{0.07,0.75}
	$\left\{p_{min},p_{max}\right\}$	{0.5,1.5}
EHO	$B_{r}$	0.2
CMA-ES	$\mu ,\omega_{i=1\ldots \mu },\sigma ,c_{\sigma },d_{\sigma },c_{c},c_{1}$	Default value

**Table 11:** Results of comparative experiments on various-scale instances.

Algorithm		IDBO	DBO	GWO	GA	PSO	WOA	WSO	EHO	CMA-ES
C10	AFV	290.5	309.1	309.6	328.3	299.7	319.8	296.1	297.3	316.7
	Gap	-	6.0%	6.2%	11.5%	3.1%	9.2%	1.9%	2.3%	8.3%
	*p*-value	1	3.95 × 10^−5^	8.79 × 10^−5^	2.55 × 10^−6^	9.77 × 10^−4^	2.52 × 10^−6^	4.89 × 10^−4^	2.44 × 10^−4^	2.51 × 10^−6^
	CT(s)	3.03	0.98	0.91	0.88	0.75	0.42	0.94	1.02	0.84
C20	AFV	591.2	712.3	624.8	691.1	657.5	685.2	682.1	642.2	661.3
	Gap	-	17.0%	5.4%	14.5%	10.1%	13.7%	13.3%	7.9%	10.6%
	*p*-value	1	1.73 × 10^−6^	5.79 × 10^−5^	1.92 × 10^−6^	2.88 × 10^−6^	2.35 × 10^−6^	1.73 × 10^−6^	2.12 × 10^−6^	1.73 × 10^−6^
	CT(s)	4.92	2.47	1.78	1.57	0.94	0.69	1.64	1.97	1.49
C30	AFV	932.3	1158	1047.4	1161.8	1139.8	1118.9	1198.5	1134.4	1083.7
	Gap	-	19.5%	11.0%	19.8%	18.2%	16.7%	22.2%	17.8%	14.0%
	*p*-value	1	1.73 × 10^−6^	2.35 × 10^−6^	1.73 × 10^−6^	1.73 × 10^−6^	1.92 × 10^−6^	1.73 × 10^−6^	1.73 × 10^−6^	1.73 × 10^−6^
	CT(s)	8.03	3.28	3.1	2.11	1.08	0.96	2.14	3.04	2.37
C40	AFV	1078.7	1475.3	1277.9	1484.2	1476.2	1436.2	1567.5	1482.3	1297.2
	Gap	-	26.9%	15.6%	27.3%	26.9%	24.9%	31.2%	27.2%	16.8%
	*p*-value	1	1.73 × 10^−6^	1.92 × 10^−6^	1.73 × 10^−6^	1.73 × 10^−6^	1.73 × 10^−6^	1.73 × 10^−6^	1.73 × 10^−6^	1.73 × 10^−6^
	CT(s)	15.29	3.62	4.41	2.8	1.42	1.31	2.97	3.34	3.08
C50	AFV	1472.5	1877.8	1646.4	1996	1944	1779	2015.6	1989.5	1739.1
	Gap	-	21.6%	10.6%	26.2%	24.3%	17.2%	26.9%	26.0%	15.3%
	*p*-value	1	1.73 × 10^−6^	4.29 × 10^−6^	1.73 × 10^−6^	1.73 × 10^−6^	1.73 × 10^−6^	1.73 × 10^−6^	1.73 × 10^−6^	1.73 × 10^−6^
	CT(s)	23.7	4.63	5.34	3.5	1.74	1.6	3.16	3.49	3.53
C60	AFV	1945.7	2488.3	2159.8	2571.5	2538.5	2317.8	2634.9	2634.9	2205.8
	Gap	-	21.8%	9.9%	24.3%	23.4%	16.1%	26.2%	26.2%	11.8%
	*p*-value	1	1.73 × 10^−6^	8.9 × 10^−5^	1.73 × 10^−6^	1.73 × 10^−6^	1.73 × 10^−6^	1.73 × 10^−6^	1.73 × 10^−6^	1.73 × 10^−6^
	CT(s)	74.26	7.54	8.83	6.52	2.01	2.45	4.59	5.06	4.29
Performance		~	+	+	+	+	+	+	+	+

**Table 12:** Results of comparative experiments on various-distribution instances.

Algorithm		IDBO	DBO	GWO	GA	PSO	WOA	WSO	EHO	CMA-ES
D30-1	AFV	927.3	1145.4	995.7	1128.8	1091.6	1082.8	1109.2	1090.1	998.2
	Gap	-	19.0%	6.9%	17.9%	15.1%	14.4%	16.4%	14.9%	7.1%
	*p*-value	1	1.73 × 10^−6^	1.97 × 10^−5^	1.73 × 10^−6^	1.73 × 10^−6^	1.92 × 10^−6^	1.73 × 10^−6^	1.73 × 10^−6^	1.92 × 10^−6^
D30-2	AFV	973.1	1156.2	1015.5	1192.2	1123	1130.91	1147.8	1137.3	1045.77
	Gap	-	15.8%	4.2%	18.4%	13.3%	14.0%	15.2%	14.4%	6.9%
	*p*-value	1	1.73 × 10^−6^	1.6 × 10^−4^	1.73 × 10^−6^	1.73 × 10^−6^	1.73 × 10^−6^	1.73 × 10^−6^	1.73 × 10^−6^	4.29 × 10^−6^
D30-3	AFV	957.8	1235	1011.6	1198.1	1126.4	1148.6	1144.3	1122.2	1023
	Gap	-	22.4%	5.3%	20.1%	15.0%	16.6%	16.3%	14.6%	6.4%
	*p*-value	1	1.73 × 10^−6^	6.39 × 10^−4^	1.92 × 10^−6^	1.73 × 10^−6^	1.73 × 10^−6^	1.73 × 10^−6^	1.73 × 10^−6^	1.6 × 10^−6^
P30-1	AFV	865.7	1047.5	908.1	1078.7	924.1	1019.4	934.3	928.4	925.23
	Gap	-	17.4%	4.7%	19.7%	6.3%	15.1%	7.3%	6.8%	6.4%
	*p*-value	1	1.73 × 10^−6^	2.84 × 10^−5^	1.73 × 10^−6^	2.6 × 10^−6^	1.73 × 10^−6^	2.6 × 10^−6^	7.7 × 10^−6^	1.5 × 10^−5^
P30-2	AFV	884.4	1174	973.5	1156	1111.5	1118.2	1171.4	1084.9	999.9
	Gap	-	24.7%	9.2%	23.5%	20.4%	20.9%	24.5%	18.5%	11.6%
	*p*-value	1	1.73 × 10^−6^	1.8 × 10^−5^	1.73 × 10^−6^	1.73 × 10^−6^	1.92 × 10^−6^	1.73 × 10^−6^	1.73 × 10^−6^	1.92 × 10^−6^
P30-3	AFV	1103.3	1341	1189.2	1325.7	1282	1256	1331.2	1277.5	1226.1
	Gap	-	17.7%	7.2%	16.8%	13.9%	12.2%	17.1%	13.6%	10.0%
	*p*-value	1	1.73 × 10^−6^	3.02 × 10^−6^	1.73 × 10^−6^	1.73 × 10^−6^	1.73 × 10^−6^	1.73 × 10^−6^	1.73 × 10^−6^	1.73 × 10^−6^
Performance		~	+	+	+	+	+	+	+	+

## Data Availability

The authors can provide the data used in this study upon request.
